# Design, synthesis and biological evaluation of novel diarylpyridine derivatives as tubulin polymerisation inhibitors

**DOI:** 10.1080/14756366.2022.2130284

**Published:** 2022-10-05

**Authors:** Shanbo Yang, Chao Wang, Lingyu Shi, Jing Chang, Yujing Zhang, Jingsen Meng, Wenjing Liu, Jun Zeng, Renshuai Zhang, Yingchun Shao, Dongming Xing

**Affiliations:** a The Affiliated Hospital of Qingdao University, Cancer Institute, School of Basic Medicine, Qingdao University, Qingdao, China; b Qingdao Cancer Institute, Qingdao, China; c The Affiliated Cardiovascular Hospital of Qingdao University, Qingdao University, Qingdao, China; d School of Life Sciences, Tsinghua University, Beijing, China

**Keywords:** Diarylpyridine, antiproliferative activity, colchicine binding site inhibitor, tubulin

## Abstract

A set of novel diarylpyridines as anti-tubulin agents were designed, synthesised using a rigid pyridine as a linker to fix the *cis*-orientation of ring-A and ring-B. All of the target compounds were evaluated for their *in vitro* antiproliferative activities. Among them, **10t** showed remarkable antiproliferative activities against three cancer cell lines (HeLa, MCF-7 and SGC-7901) in sub-micromolar concentrations. Consistent with its potent antiproliferative activity, **10t** also displayed potent anti-tubulin activity. Cellular mechanism investigation elucidated **10t** disrupted the cellular microtubule structure, arrested cell cycle at G2/M phase and induces apoptosis. Molecular modelling studies showed that **10t** could bind to the colchicine binding site on microtubules. These results provide motivation and further guidance for the development of new CA-4 analogues.

## Introduction

Microtubules are hollow tubular structures composed of heterodimers of *α*-tubulin and *β*-tubulin, which have a variety of roles in eukaryotic cells, including maintenance of cell morphology, cell growth, cell motility, material transport, organelle transport, signalling, mitosis, etc.^1–3^ If the dynamic cycle of microtubule assembly–disassembly is disrupted, the mitotic process of tumour cells is affected, thereby inhibiting their growth and leading to apoptosis.^4^ Therefore, drugs that interfere with the kinetics of microtubule protein depolymerisation and polymerisation are an important class of antitumor drugs.^5^ Several clinical agents have been developed (e.g. paclitaxel and vincristine), but there are currently no FDA-approved inhibitors of microtubulin at the colchicine site. The development of microtubulin polymerisation inhibitors targeting the colchicine binding site has, therefore, attracted the interest of many medicinal chemists.^6^

**Figure 1.**
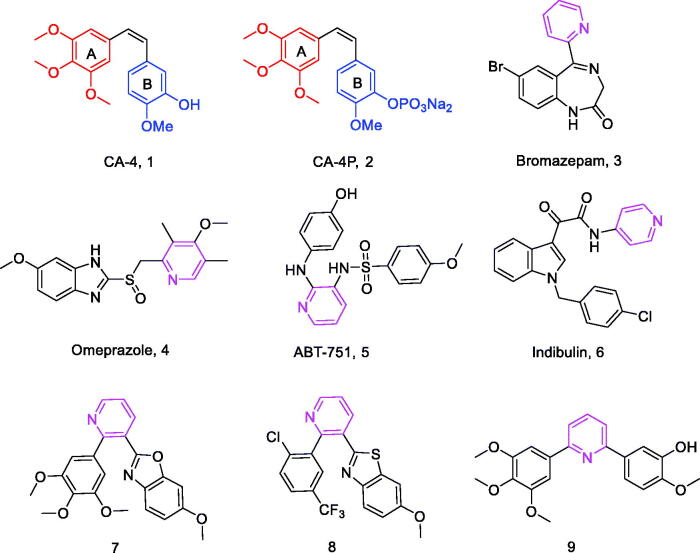
Chemical structures of CA-4, CA-4P, and some pyridine derivatives.

Combretastatin A-4 (**1**, Figure 1) is a natural product, first extracted from the bark of the South African willow tree *Combretum caffrum* in 1989, that inhibits tubulin polymerisation by interacting with colchicine binding site on tubulin.^7^ This *cis*-stilbene shows excellent cytotoxicity against a wide range of human cancer cell lines, including multidrug-resistant cancer cell lines.^8^^,^^9^ CA-4P (**2**, Figure 1), its soluble prodrug, is currently under clinical investigation as a combination therapy for various multidrug-resistant solid tumours.^10^ Due to the structural simplicity of CA-4, numerous structure-activity relationships (SAR) studies have been performed on this compound and its analogs by many academic and industrial groups. SAR studies have shown that the *cis*-orientation of the double bond and the presence of 3,4,5-trimethoxyphenyl as ring A are essential to produce potent potency.^11^ A ring is an essential requirement for potent cytotoxicity. Unfortunately, CA-4 and other olefinic analogs are prone to isomerise to inactive trans-forms during storage and administration.^12^ In order to avoid the stability problems of CA-4, the olefinic groups of the A and B rings are fixed by introducing various cyclic structures such as three, five and six-membered rings.^13–15^

Pyridine is a six-membered aromatic heterocycle containing a nitrogen atom, and its derivatives (**3–6**, Figure 1) have been of interest because of its ease of preparation and many potential pharmacological properties,^16^ including anxiolytic,^17^ anti-ulcer^18^ and anti-tubulin activities.^19^ For example, a series of substituted pyridine compounds have been reported as tubulin polymerisation inhibitors (**7–9**, Figure 1).^20–22^

**Figure 2.**
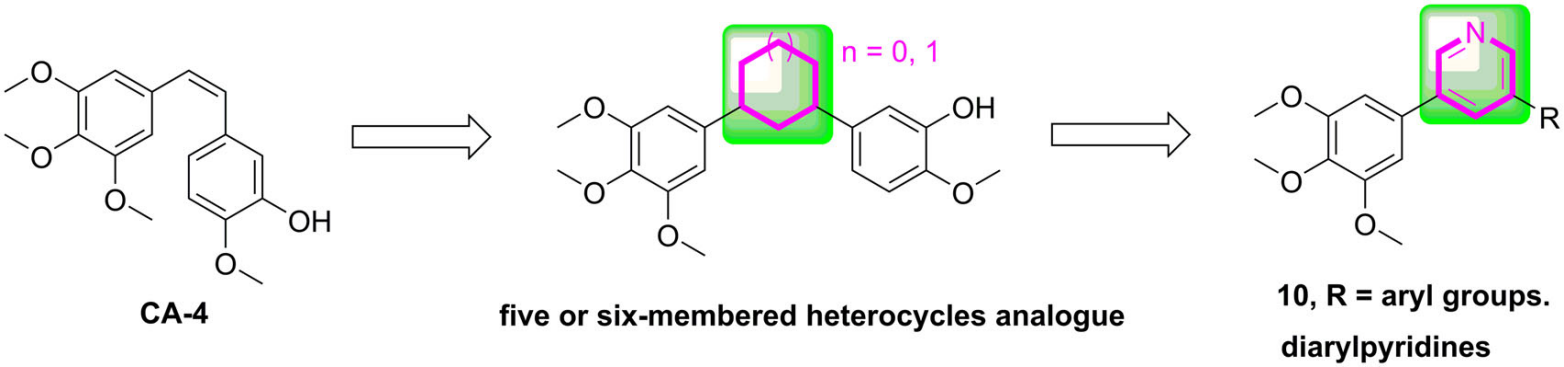
The rational design of target compounds.

In our study, pyridine fragment was introduced into the CA-4 skeleton to replace the olefinic group between the A and B rings. As a result, a series of diarylpyridines derivatives (**10**, Figure 2) were designed and synthesised. The newly synthesised target compounds were investigated for their biological activities to explore preliminary SAR and molecular modelling was performed to elucidate their possible binding modes in tubulin.

## Results and discussion

### Chemistry

**Scheme 1.**
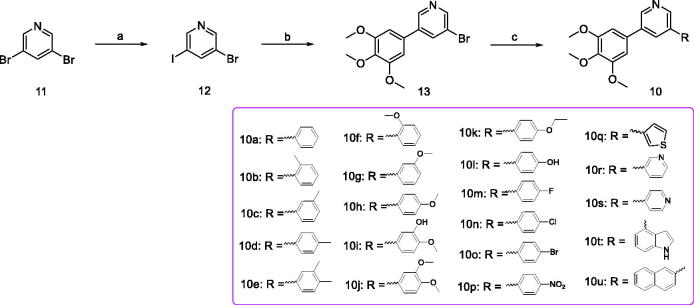
Reagents and conditions (a) *iPr*MgCl^.^LiCl, iodine, piperidine, THF, –10 to 5 °C, 10 min; (b) 3,4,5-trimethoxybenzeneboronic acid, Pd(PPh_3_)_4_, K_2_CO_3_, 1,4-dioxane/H_2_O = 3/1, N_2_ atmosphere, 126 °C, M.W., 25 min; (c) substituted phenylboronic acid, Pd(PPh_3_)_4_, K_2_CO_3_, 1,4-dioxane/H_2_O = 3/1, N_2_ atmosphere, 126 °C, M.W., 25 min.

The synthetics of target compounds **10a-10u** was shown in Scheme 1. Firstly, the 3-bromo-5-iodopyridine (**12**) was synthesised by using 3,5 dibromo-pyridine (**11**) as the starting material.^23^ Subsequently, **12** reacted with 3,4,5-trimethoxybenzeneboronic acid, Pd(PPh_3_)_4_ and K_2_CO_3_ in 1,4-dioxane/H_2_O to afford 3-bromo-5–(3,4,5-trimethoxyphenyl)pyridine (**13**).^24^ Lastly, **13** was reacted with the corresponding phenylboronic acid *via* Suzuki crosscoupling reaction to generate target compounds **10a-10u.**^25^

### Biological evaluation

#### In vitro antiproliferative activity

The antiproliferative activities of diarylpyridines **10a-10u** were evaluated against human cancer cell lines, namely cervical (HeLa), gastric adenocarcinoma (SGC-7901) and breast (MCF-7), respectively, using the standard MTT assay with CA-4 as positive control. As illustrated in Table 1, some target compounds exhibited moderate to potent antiproliferative activities. Among them, **10t**, which contained an indole moiety as the B ring, displayed the most potent antiproliferative activities against HeLa, SGC-7901 and MCF-7 cell lines with IC_50_ values of 0.19, 0.30 and 0.33 μM, respectively.

**Table 1.** Antiproliferative activity of all compounds.

Compounds	(IC_50_ ± SD, μM)^a^		
	HeLa	SGC-7901	MCF-7
**10a**	20.21 ± 2.1	30.57 ± 2.5	39.83 ± 4.7
**10b**	50.62 ± 4.7	55.31 ± 5.6	>60
**10c**	3.28 ± 0.27	5.24 ± 0.69	8.49 ± 0.86
**10d**	15.22 ± 1.2	16.58 ± 2.2	13.94 ± 1.9
**10e**	>60	>60	>60
**10f**	37.83 ± 3.6	>60	25.80 ± 2.7
**10g**	17.78 ± 1.0	18.91 ± 0.97	>60
**10h**	4.20 ± 0.8	9.73 ± 1.7	8.54 ± 0.86
**10i**	13.74 ± 1.2	14.32 ± 1.1	19.58 ± 2.2
**10j**	>60	>60	>60
**10k**	39.63 ± 4.1	>60	>60
**10l**	18.81 ± 1.9	16.88 ± 1.7	29.61 ± 2.8
**10m**	>60	>60	>60
**10n**	30.83 ± 4.1	>60	>60
**10o**	>60	>60	>60
**10p**	>60	40.10 ± 5.2	>60
**10q**	>60	>60	>60
**10r**	55.21 ± 6.6	49.81 ± 5.1	>60
**10s**	>60	>60	>60
**10t**	**0.19 ± 0.013**	**0.30 ± 0.030**	**0.33 ± 0.028**
**10u**	>60	>60	>60
**CA-4** ^b^	0.05 ± 0.004	0.08 ± 0.006	0.04 ± 0.002

Bold represents the IC_50_ value of the target compound with the best activity.

^a^IC_50_: the half maximal inhibitory concentration.

^b^Used as positive controls.

The SAR of the 21 target compounds have been summarised. Firstly, a sharply decline of antiproliferative activity was observed when other aryl groups such as thiophene (**10q**), pyridine (**10r** and **10s**) and naphthalene (**10u**) were introduced to replace the B ring. Furthermore, when the B ring is benzene, **10a** with the unsubstituted B ring displayed moderate active, the introduction of electron donating groups on B ring, such as -CH_3_ (**10c**, **10d**), -OCH_3_ (**10g**, **10h**), -OCH_2_CH_3_ (**10k**), −3-OH-4-OCH_3_ (**10i**), resulted in maintenance or increase in antiproliferative activity. However, the antiproliferative activity was decreased when introduced electron withdrawing groups on B-ring, such as, -F (**10m**), -Cl (**10n**), -Br (**10o**) and -NO_2_ (**10p**). Furthermore, the simultaneous introduction of -CH_3_ (**10e**) and -OCH_3_ (**10j**) at the 3,4-position of the B-ring resulted in drastically decreased inhibitory activity, probably due to two methoxy groups are bulky and have steric hindrance.

#### Tubulin polymerisation

**Figure 3.**
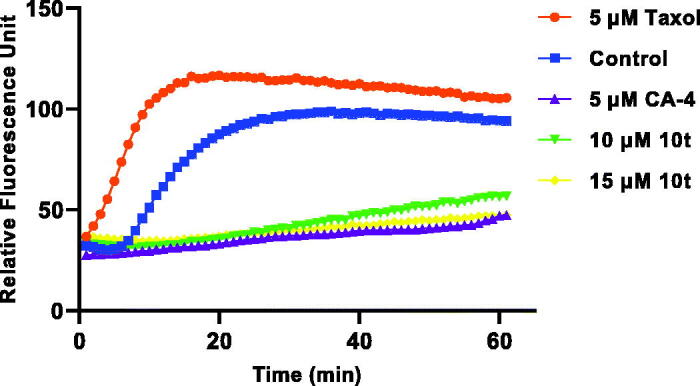
Effect of **10t** on tubulin polymerisation. Tubulin had been pre-incubated for 1 min with **10t** at 10 and 15 μM, CA-4 at 5 μM, taxol at 5 μM or vehicle DMSO at room temperature before GTP was added to start the tubulin polymerisation reactions. The reaction was monitored at 37 °C.

CA-4 binds to microtubule heterodimers, thereby inhibiting microtubule polymerisation. In order to investigate whether the target compound **10t** act on the microtubule system, **10t** were selected and compared with the positive control drug CA-4 and the negative control drug taxol on the inhibition of tubulin polymerisation. As shown in Figure 3, **10t** significantly inhibited tubulin polymerisation compared with the negative control drug, taxol. **10t** inhibited tubulin polymerisation in a dose-dependent manner. The results showed that **10t** act on the tubulin system and interfere with tubulin polymerisation.

#### Analysis of immunofluorescence staining

**Figure 4.**
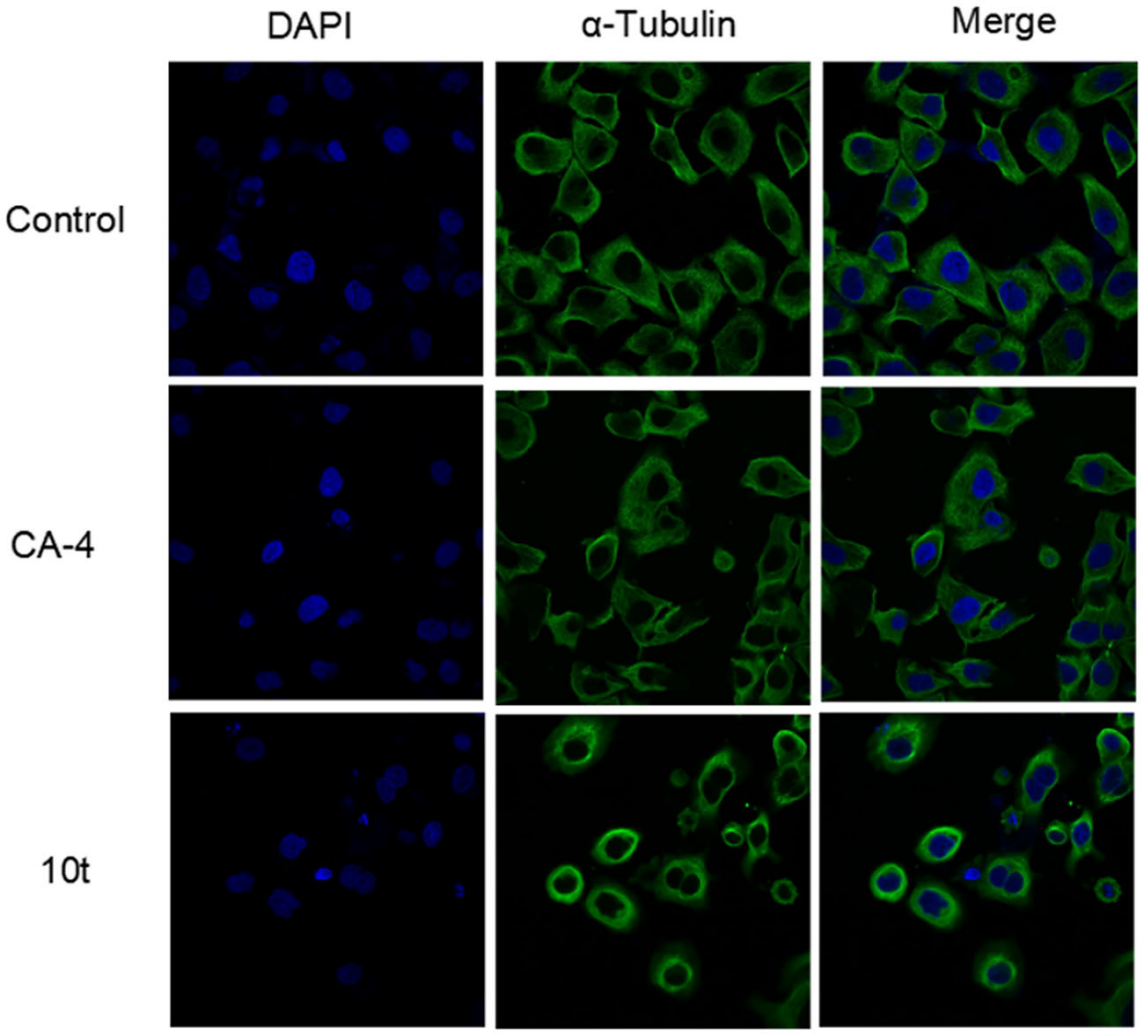
Effects of 2-fold IC_50_ CA-4 and 2-fold IC_50_ **10t** on the cellular microtubule networks of Hela cells by immunofluorescence assay. Microtubules and unassembled microtubule proteins stained with *α*-tubulin primary antibody and FITC secondary antibody, shown in green, and nuclei stained with DAPI, shown in the blue colour.

Microtubule inhibitors can inhibit the formation of spindle filaments, lead to improper chromosome segregation, hinder the division process after metaphase, and which can have some effect on the cytoskeleton. To further determine the effect of target compound **10t** on tubulin, immunofluorescence experiments were used to observe the effects of 2-fold IC_50_ **10t** and 2-fold IC_50_ CA-4 on the microtubule network on Hela cells. As shown in Figure 4, untreated cells exhibited normal arrangement and organisation of microtubules. When treated with the **10t** and CA-4, microtubules became shorter and wrapped around the nucleus compared to the control group. The results showed that the **10t** exhibited similar characteristics to CA-4 and could disrupt the microtubule network and destabilise the microtubules.

#### Analysis of cell cycle

**Figure 5.**
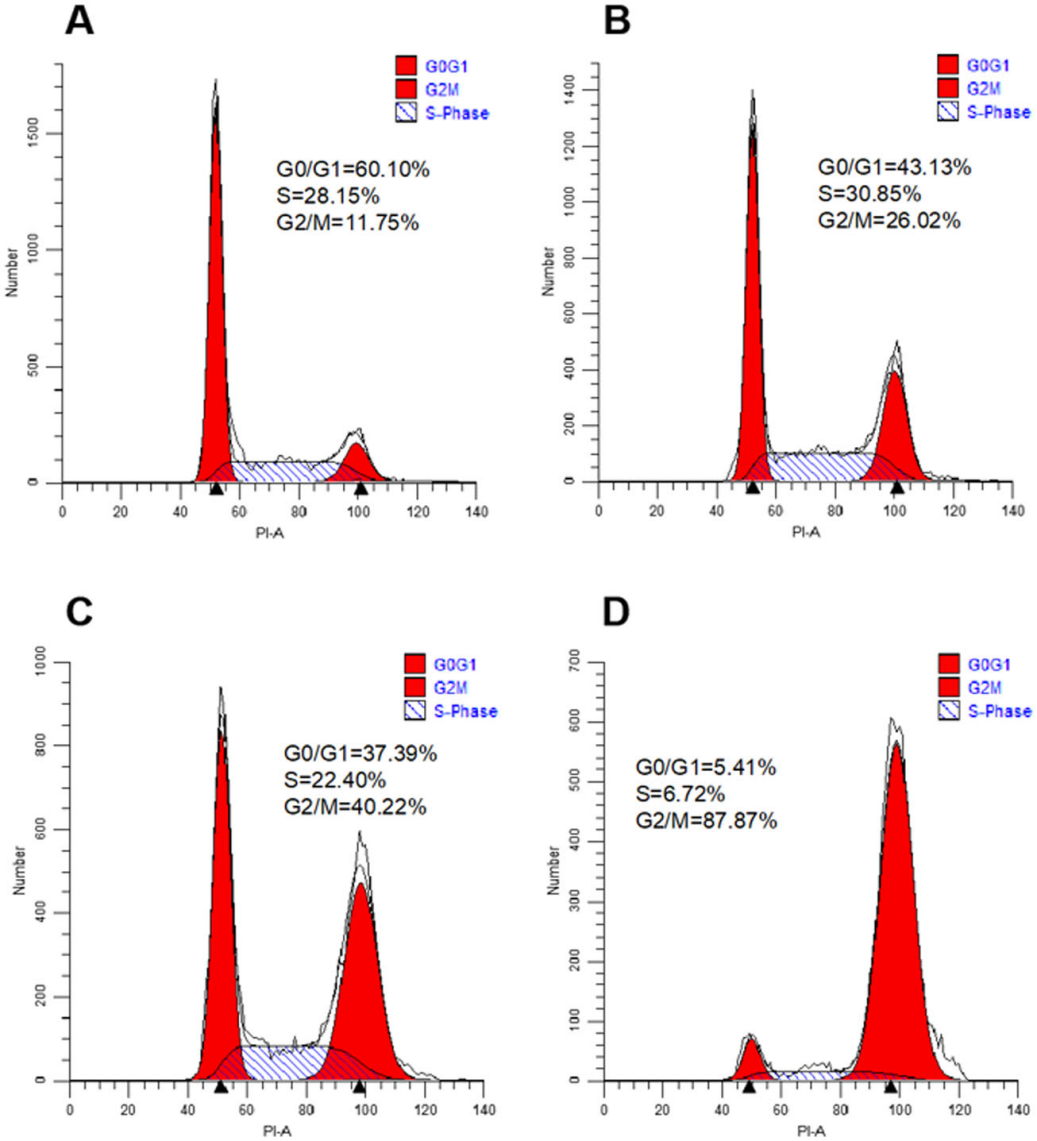
Cell cycle distribution of Hela cells after 24 h treatment with 1-fold IC_50_, 2-fold IC_50_ and 3-fold IC_50_ of **10t**. (A) Control, (B) 1-fold IC_50_, (C) 2-fold IC_50_ and (D) 3-fold IC_50_.

To assess the effect of target compounds on mitosis, the effect of **10t** on Hela cell cycle progression was next investigated by flow cytometry. In this study, Hela cells were treated with increasing doses of **10t** for 24 h. As shown in Figure 5, it was found that the **10t** inhibited the cells at 1-fold IC_50_, 2-fold IC_50_ and 3-fold IC_50_, respectively. The proportions of cells blocked in G2/M phase were 26.02%, 40.22% and 87.87%, while the proportion of untreated control cells in G2/M phase was 11.75%. **10t** was demonstrated to clearly cause G2/M phase arrest in a time-dependent manner.

#### Analysis of cell apoptosis

**Figure 6.**
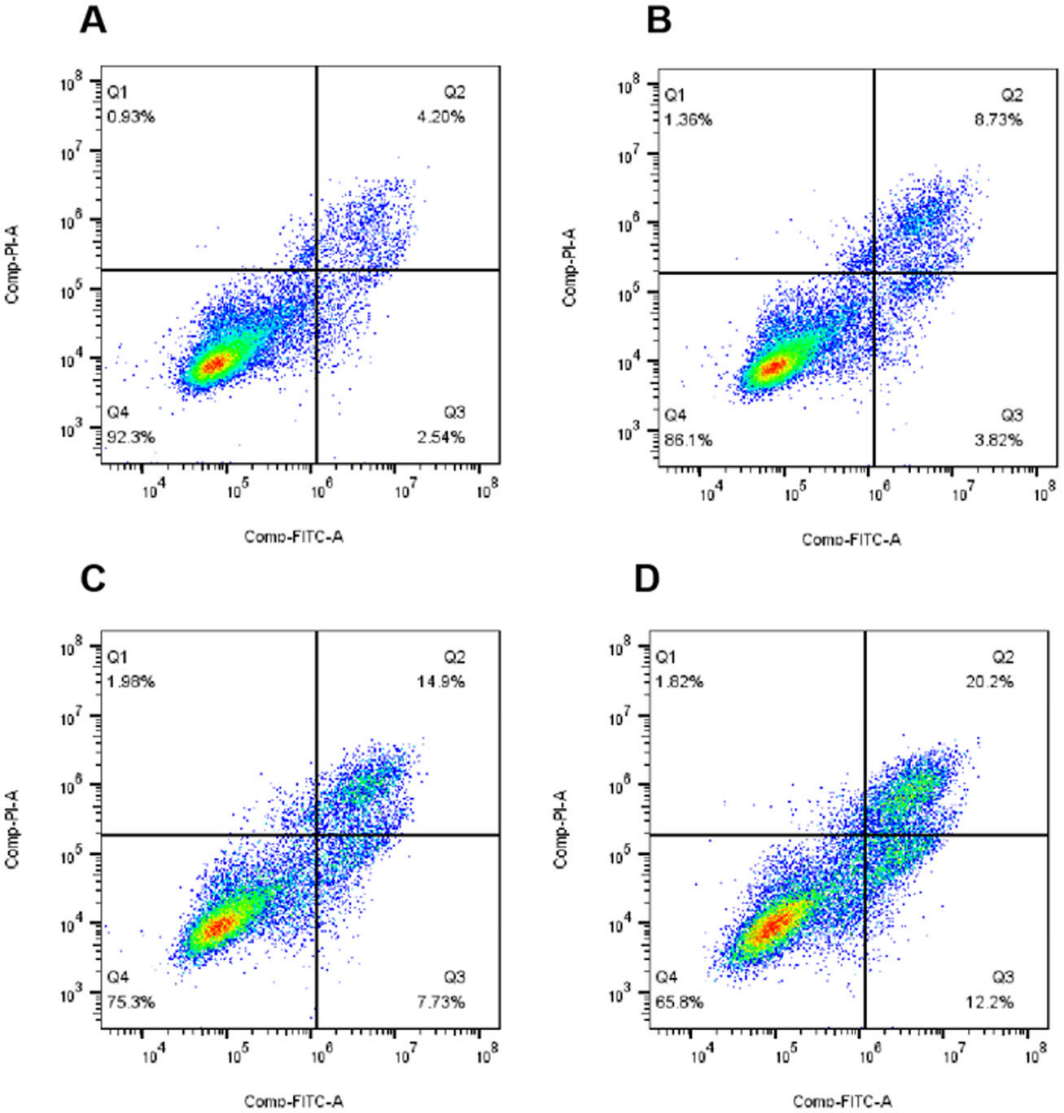
Proportion of apoptotic cells in Hela cells after 48 h treatment with 1-fold IC_50_, 2-fold IC_50_ and 3-fold IC_50_ of **10t**. (A) Control, (B) 1-fold IC_50_, (C) 2-fold IC_50_ and (D) 3-fold IC_50_.

Appearance of cell cycle arrest leads to apoptosis. To assess whether the target compounds can induce apoptosis, **10t** were detected on Hela cells using Annexin V-FITC/PI. As shown in Figure 6, it was found that the proportion of apoptotic cells in the untreated control group was 7.67%. After treatment with 1-fold IC_50_, 2-fold IC_50_ and 3-fold IC_50_, the proportions of apoptotic cells were 13.91%, 24.61% and 34.22%. The results of apoptosis showed that **10t** induced Hela apoptosis in a dose-dependent manner.

#### Molecular modelling study

**Figure 7.**
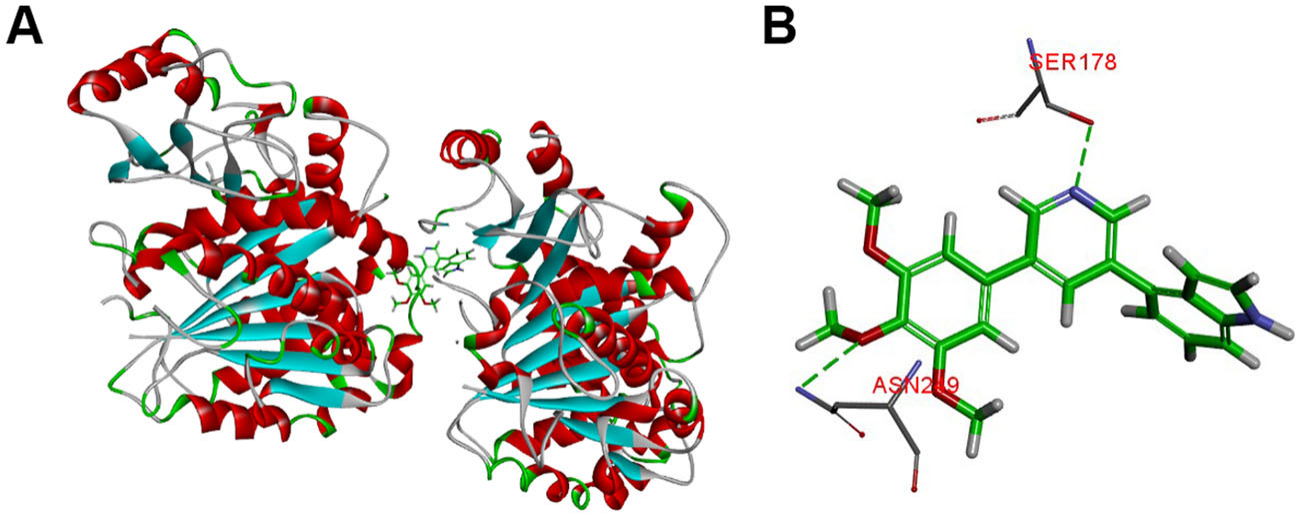
(A) The binding mode of compound **10t** in the colchicine binding site of tubulin; (B) overlay of **10t** in the binding site.

The representative compound **10t** was firstly selected to explain the reasons for the variability in the potency *via* molecular docking analysis with tubulin crystal structure (PDB: 1SA0) using the CDOCKER program of Discovery Studio 3.0 software. As given in Figure 7, the 3,4,5-trimethoxybenzoyl A ring of **10t** was located deeply into the *β*-subunit of tubulin. The B ring of **10t** extends towards the *α*/*β*-tubulin interface and several important amino acids of tubulin formed hydrogen bond interactions with **10t**. The residue of *β*-ASN249 forms a hydrogen bond with the oxygen of the methoxy group (A ring). Furthermore, nitrogen atom of pyridine forms a hydrogen bond with the residue *β*-SER178. The docking results suggested that **10t** may exhibit its biological activities by binding to colchicine site.

## Conclusion

In summary, we replaced the double bond linker between the A and B rings by introducing a pyridine fragment into the CA-4 skeleton. We developed a series of diarylpyridines and evaluated their antiproliferative activity and tubulin polymerisation inhibition. Some synthesised compounds displayed moderate to potent antiproliferative activities with IC_50_ values at the sub-micromolar level. Among compounds, the existence of indole group was the main factor affecting the biological activity. **10t** has broad-spectrum antitumor activity against all tumour cell lines tested with IC_50_ value of 0.19–0.33 μM. Consistent with its antiproliferative activity, **10t** also exhibited potent antitubulin activity, similar to that of CA-4. Further mechanistic studies confirmed that **10t** was a microtubule-destabilizing agent that induced the accumulation of cells in the G2/M phase, caused cell mitotic catastrophe and apoptosis. Additionally, the results of docking study showed that **10t** may bind to colchicine binding site of tubulin. Our work not only expands the exploration of the linker modification of tubulin inhibitor CA-4 but also provides a set of rigid analogues with moderate to potent antiproliferative activity.

## Experiment section

### Chemistry

#### Materials and methods

All reagents and solvents were obtained from commercial sources. The reaction was monitored by TLC with silica gel plates under ultraviolet (UV) light (wavelength: 365 nm and 254 nm). ^1^H (500 MHz) and ^13^C NMR (125 MHz) were measured on an Agilent ProPulse 500 MHz at room temperature using CDCl_3_ as solvent. High-resolution mass spectra (HRMS) were recorded by Agilent Accurate-Mass Q-TOF 6530 instrument in ESI mode. The microwave reactions were carried out in a single mode cavity microwave synthesiser (CEM Corporation, NC, USA).

#### General synthetic procedures for 3-bromo-5-iodopyridine (12)

To a solution of 3,5dibromo-pyridine (5.4 mmol) in THF (25 ml), was added a solution of *iPr*MgCl_._LiCl (5.9 mmol, 1.3 M in THF) at −10 °C. After 10 min of stirring a solution of iodine (5.9 mmol) in THF (10 ml) was added dropwise, keeping the temperature below −5 °C. After 10 min of stirring Et_2_O (100 ml) was added and was washed with NaHSO_3_ sat. (30 ml). Organic extract was washed with brine (50 ml), dried over Na_2_SO_4_, filtered and concentrated to give the desired product without further necessary purification.

#### General synthetic procedures for 3-bromo-5-(3,4,5-trimethoxyphenyl)pyridine (13)

A mixture of **12** (0.40 mmol), Pd(PPh_3_)_4_ (0.04 mmol), and K_2_CO_3_ (0.48 mmol), and 3,4,5-trimethoxybenzeneboronic acid (0.41 mmol) in 1,4-dioxane/H_2_O (15 ml, 3:1) was degassed and purged with N_2_ for about three times. After stirred at irradiated in a microwave reactor for 25 min at 130 °C (indicated by TLC) under N_2_ atmosphere, the reaction mixture was poured into H_2_O (50 ml) and extracted with ethyl acetate (80 ml × 3). The combined organics were washed with brine (10 ml × 3), dried over anhydrous Na_2_SO_4_, filtered and concentrated under vacuum to give a residue, which was purified by column 300 chromatography using a mixture of petroleum ether and ethyl acetate (3:1) as an eluent to provide the target compound **13** in yields of 80%.

#### General synthetic procedures for diarylpyridines (10)

A mixture of **13** (0.10 mmol), Pd(PPh_3_)_4_ (0.01 mmol), and K_2_CO_3_ (0.12 mmol), and substituted phenylboronic acid (0.11 mmol) in 1,4-dioxane/H_2_O (5 ml, 3:1) was degassed and purged with N_2_ for about three times. After stirred at irradiated in a microwave reactor for 25 min at 130 °C (indicated by TLC) under N_2_ atmosphere, the reaction mixture was poured into H_2_O (50 ml) and extracted with ethyl acetate (80 ml × 3). The combined organics were washed with brine (10 ml × 3), dried over anhydrous Na_2_SO_4_, filtered and concentrated under vacuum to give a residue, which was purified by column 300 chromatography using a mixture of petroleum ether and ethyl acetate (3:1) as an eluent to provide the target compounds (***10a-10u***) in yields of 42–95%.

##### 3-Phenyl-5-(3,4,5-trimethoxyphenyl)pyridine (10a)

White solid; yield: 42%; m.p. 116–117 °C; ^1^H NMR (500 MHz, CDCl_3_) δ 8.89 (s, 2H), 8.09 (s, 1H), 7.64 (d, *J* = 7.3 Hz, 2H), 7.53 (t, *J* = 7.5 Hz, 2H), 7.47 (t, *J* = 7.3 Hz, 1H), 6.80 (s, 2H), 3.94 (s, 6H), 3.92 (s, 3H); ^13^C NMR (125 MHz, CDCl_3_) δ 153.89 (2C), 144.93 (2C), 138.64, 134.11, 133.64 (2C), 129.67, 129.28 (2C), 128.69, 127.56, 127.30 (2C), 104.62 (2C), 61.00, 56.32 (2C); HRMS calcd for C_20_H_20_NO_3_ [M + H]^+^ 322.1443, found 322.1439.

##### 3-(O-Tolyl)-5-(3,4,5-trimethoxyphenyl)pyridine (10b)

White solid; yield: 89%; m.p. 84–86 °C; ^1^H NMR (500 MHz, CDCl_3_) δ 8.83 (s, 1H), 8.59 (s, 1H), 7.84 (s, 1H), 7.36–7.32 (m, 2H), 7.32–7.27 (m, 2H), 6.80 (s, 2H), 3.93 (s, 6H), 3.91 (s, 3H), 2.32 (s, 3H); ^13^C NMR (125 MHz, CDCl_3_) δ 153.82 (2C), 148.01, 145.97, 138.46, 137.70, 135.65, 135.16, 133.21, 130.68 (2C), 129.89, 128.37, 126.19, 124.82, 104.50 (2C), 60.98, 56.28 (2C), 20.42; HRMS calcd for C_21_H_22_NO_3_ [M + H]^+^ 336.1600, found 336.1604.

##### 3-(M-Tolyl)-5-(3,4,5-trimethoxyphenyl)pyridine (10c)

White solid; yield: 74%; m.p. 80–81 °C; ^1^H NMR (500 MHz, CDCl_3_) δ 8.83 (s, 2H), 7.99 (s, 1H), 7.44 (d, *J* = 7.0 Hz, 2H), 7.40 (t, *J* = 7.9 Hz, 1H), 7.26 (d, *J* = 5.6 Hz, 1H), 6.81 (s, 2H), 3.95 (s, 6H), 3.91 (s, 3H), 2.46 (s, 3H); ^13^C NMR (125 MHz, CDCl_3_) δ 153.78 (2C), 146.88, 146.68, 138.87 (2C), 138.39, 137.69, 133.65, 132.86, 129.05 (2C), 128.02, 124.40, 109.99, 104.64 (2C), 60.98, 56.32 (2C), 21.52; HRMS calcd for C_21_H_22_NO_3_ [M + H]^+^ 336.1600, found 336.1601.

##### 3-(P-Tolyl)-5-(3,4,5-trimethoxyphenyl)pyridine (10d)

White solid; yield: 55%; m.p. 152–153 °C; ^1^H NMR (500 MHz, CDCl_3_) δ 8.80 (s, 2H), 7.99 (s, 1H), 7.54 (d, *J* = 8.1 Hz, 2H), 7.32 (d, *J* = 7.9 Hz, 2H), 6.80 (s, 2H), 3.95 (s, 6H), 3.91 (s, 3H), 2.43 (s, 3H); ^13^C NMR (125 MHz, CDCl_3_) δ 153.78 (2C), 146.57, 146.34, 138.39, 138.33 (2C), 134.70, 133.59, 132.78 (2C), 129.87 (2C), 127.10 (2C), 104.61 (2C), 60.98, 56.31 (2C), 21.17; HRMS calcd for C_21_H_22_NO_3_ [M + H]^+^ 336.1600, found 336.1597.

##### 3-(3,4-Dimethylphenyl)-5-(3,4,5-trimethoxyphenyl)pyridine (10e)

White solid; yield: 80%; m.p. 124–125 °C; ^1^H NMR (500 MHz, CDCl_3_) δ 8.79 (d, *J* = 18.9 Hz, 2H), 7.98 (t, *J* = 2.0 Hz, 1H), 7.41 (s, 1H), 7.38 (d, *J* = 7.7 Hz, 1H), 7.27 (d, *J* = 7.8 Hz, 1H), 6.80 (s, 2H), 3.95 (s, 6H), 3.91 (s, 3H), 2.37 (s, 3H), 2.34 (s, 3H); ^13^C NMR (125 MHz, CDCl_3_) δ 153.77 (2C), 146.59, 146.24, 138.38, 137.47, 137.00, 136.89, 136.80, 135.13, 133.63, 132.84, 130.42, 128.44, 124.62, 104.63 (2C), 60.98, 56.31 (2C), 19.92, 19.50; HRMS calcd for C_22_H_24_NO_3_ [M + H]^+^ 350.1756, found 350.1753.

##### 3-(2-Methoxyphenyl)-5-(3,4,5-trimethoxyphenyl)pyridine (10f)

White solid; yield: 75%; m.p. 78–80 °C; ^1^H NMR (500 MHz, CDCl_3_) δ 8.75 (s, 2H), 8.00 (s, 1H), 7.44–7.33 (m, 2H), 7.09 (t, *J* = 7.5 Hz, 1H), 7.04 (d, *J* = 8.2 Hz, 1H), 6.80 (s, 2H), 3.94 (s, 6H), 3.91 (s, 3H), 3.84 (s, 3H); ^13^C NMR (125 MHz, CDCl_3_) δ 156.61, 153.72 (2C), 148.85, 146.18, 138.22, 135.27, 133.80, 130.70 (2C), 129.76, 126.80, 121.10 (2C), 111.32, 104.60 (2C), 60.97, 56.26 (2C), 55.57; HRMS calcd for C_21_H_22_NO_4_ [M + H]^+^ 352.1549, found 352.1547.

##### 3-(3-Methoxyphenyl)-5-(3,4,5-trimethoxyphenyl)pyridine (10g)

White solid; yield: 43%; m.p. 94–96 °C; ^1^H NMR (500 MHz, CDCl_3_) δ 8.75 (s, 2H), 7.99 (s, 1H), 7.40 (td, *J* = 9.4, 1.6 Hz, 2H), 7.09 (t, *J* = 7.5 Hz, 1H), 7.04 (d, *J* = 8.2 Hz, 1H), 6.80 (s, 2H), 3.94 (s, 6H), 3.91 (s, 3H), 3.85 (s, 3H); ^13^C NMR (125 MHz, CDCl_3_) δ 156.61, 153.71 (2C), 148.95, 146.28, 138.20, 136.16, 135.22, 134.12, 133.84, 130.70, 129.73, 126.83, 121.10, 111.32, 104.59 (2C), 60.97, 56.26 (2C), 55.57; HRMS calcd for C_21_H_22_NO_4_ [M + H]^+^ 352.1549, found 352.1557.

##### 3-(4-Methoxyphenyl)-5-(3,4,5-trimethoxyphenyl)pyridine (10h)

White solid; yield: 95%; m.p. 120–121 °C; ^1^H NMR (500 MHz, CDCl_3_) δ 8.76 (d, *J* = 19.9 Hz, 2H), 7.95 (s, 1H), 7.58 (d, *J* = 8.7 Hz, 2H), 7.04 (d, *J* = 8.7 Hz, 2H), 6.80 (s, 2H), 3.94 (s, 6H), 3.91 (s, 3H), 3.87 (s, 3H); ^13^C NMR (125 MHz, CDCl_3_) δ 159.92, 153.76 (2C), 146.58, 146.24, 138.35, 136.81, 136.26, 133.68, 132.38, 130.02, 128.35 (2C), 114.62 (2C), 104.60 (2C), 60.98, 56.30 (2C), 55.40; HRMS calcd for C_21_H_22_NO_4_ [M + H]^+^352.1549, found 352.1555.

##### 2-Methoxy-5-(5-(3,4,5-trimethoxyphenyl)pyridin-3-yl)phenol (10i)

White solid; yield: 95%; m.p. 170–171 °C; ^1^H NMR (500 MHz, CDCl_3_) δ 8.76 (d, *J* = 21.9 Hz, 2H), 7.96 (s, 1H), 7.27 (d, *J* = 2.2 Hz, 1H), 7.13 (dd, *J* = 8.3, 2.2 Hz, 1H), 6.97 (d, *J* = 8.3 Hz, 1H), 6.79 (s, 2H), 3.93 (s, 9H), 3.90 (s, 3H); ^13^C NMR (125 MHz, CDCl_3_) δ 153.77 (2C), 147.39, 146.59, 146.31, 146.03, 138.37, 136.87, 136.43, 132.01, 128.54, 128.45, 118.74, 113.71, 111.40, 104.54 (2C), 60.97, 56.30 (2C), 56.04; HRMS calcd for C_21_H_22_NO_5_ [M + H]^+^ 368.1498, found 368.1498.

##### 3-(3,4-Dimethoxyphenyl)-5-(3,4,5-trimethoxyphenyl)pyridine (10j)

White solid; yield: 81%; m.p. 110–112 °C; ^1^H NMR (500 MHz, CDCl_3_) δ 8.76 (d, *J* = 17.0 Hz, 2H), 7.94 (s, 1H), 7.19 (dd, *J* = 8.3, 2.1 Hz, 1H), 7.12 (d, *J* = 2.1 Hz, 1H), 7.00 (d, *J* = 8.3 Hz, 1H), 6.80 (s, 2H), 3.96 (s, 3H), 3.94 (s, 9H), 3.91 (s, 3H); ^13^C NMR (125 MHz, CDCl_3_) δ 153.78 (2C), 149.53, 149.45, 146.75, 146.46, 138.39, 136.86, 136.52, 133.67, 132.53, 130.51, 119.78, 111.74, 110.45, 104.67 (2C), 60.98, 56.33 (2C), 56.10, 56.04; HRMS calcd for C_22_H_24_NO_5_ [M + H]^+^382.1654, found 382.1657.

##### 3-(4-Ethoxyphenyl)-5-(3,4,5-trimethoxyphenyl)pyridine (10k)

White solid; yield: 65%; m.p. 118–120 °C; ^1^H NMR (500 MHz, CDCl_3_) δ 8.76 (d, *J* = 22.4 Hz, 2H), 7.95 (s, 1H), 7.57 (d, *J* = 8.7 Hz, 2H), 7.03 (d, *J* = 8.7 Hz, 2H), 6.80 (s, 2H), 4.10 (q, *J* = 7.0 Hz, 2H), 3.95 (s, 6H), 3.91 (s, 3H), 1.46 (t, *J* = 7.0 Hz, 3H); ^13^C NMR (125 MHz, CDCl_3_) δ 159.29, 153.76 (2C), 146.59, 146.20, 138.34, 136.29, 133.72, 132.34 (2C), 129.84, 128.32 (2C), 115.15 (2C), 104.60 (2C), 63.61, 60.98, 56.31 (2C), 14.80; HRMS calcd for C_22_H_24_NO_4_ [M + H]^+^366.1705, found 366.1705.

##### 4-(5-(3,4,5-Trimethoxyphenyl)pyridin-3-yl)phenol (10l)

White solid; yield: 57%; m.p. 211–212 °C; ^1^H NMR (500 MHz, CDCl_3_) δ 8.74 (d, *J* = 22.3 Hz, 2H), 7.97 (s, 1H), 7.55 (d, *J* = 7.5 Hz, 2H), 7.04 (d, *J* = 8.5 Hz, 2H), 6.80 (s, 2H), 3.95 (s, 6H), 3.91 (s, 3H); ^13^C NMR (125 MHz, CDCl_3_) δ 157.48, 153.77 (2C), 146.16, 145.62, 138.37, 133.60, 132.12 (2C), 132.02, 128.59 (2C), 128.40, 116.35 (2C), 104.59 (2C), 60.99, 56.32 (2C); HRMS calcd for C_20_H_20_NO_4_ [M + H]^+^ 338.1392, found 338.1394.

##### 3-(4-Fluorophenyl)-5-(3,4,5-trimethoxyphenyl)pyridine (10m)

White solid; yield: 76%; m.p. 109–110 °C; ^1^H NMR (500 MHz, CDCl_3_) δ 8.78 (d, *J* = 6.6 Hz, 2H), 7.95 (s, 1H), 7.60 (dd, *J* = 8.8, 5.2 Hz, 2H), 7.20 (t, *J* = 8.6 Hz, 2H), 6.80 (s, 2H), 3.95 (s, 6H), 3.91 (s, 3H); ^13^C NMR (125 MHz, CDCl_3_) δ 163.01 (d, *J* = 248.2 Hz, 1C), 153.80 (2C), 146.94, 146.77, 138.46, 136.90, 135.72, 133.82, 133.44, 132.66, 128.96 (d, *J* = 8.2 Hz, 2C), 116.14 (d, *J* = 21.6 Hz, 2C), 104.63 (2C), 60.98, 56.32 (2C); HRMS calcd for C_20_H_19_FNO_3_ [M + H]^+^ 340.1349, found 340.1346.

##### 3-(4-Chlorophenyl)-5-(3,4,5-trimethoxyphenyl)pyridine (10n)

White solid; yield: 63%; m.p. 136–137 °C; ^1^H NMR (500 MHz, CDCl_3_) δ 8.79 (d, *J* = 9.0 Hz, 2H), 7.95 (s, 1H), 7.57 (d, *J* = 8.4 Hz, 2H), 7.48 (d, *J* = 8.5 Hz, 2H), 6.80 (s, 2H), 3.95 (s, 6H), 3.91 (s, 3H); ^13^C NMR (125 MHz, CDCl_3_) δ 153.82 (2C), 147.21, 146.71, 138.50, 136.97, 136.13, 135.49, 134.58, 133.36, 132.65, 129.35 (2C), 128.52 (2C), 104.64 (2C), 60.99, 56.33 (2C); HRMS calcd for C_20_H_19_ClNO_3_ [M + H]^+^ 356.1053, found 356.1050.

##### 3-(4-Bromophenyl)-5-(3,4,5-trimethoxyphenyl)pyridine (10o)

White solid; yield: 51%; m.p. 137–138 °C; ^1^H NMR (500 MHz, CDCl_3_) δ 8.82 (s, 2H), 8.02 (s, 1H), 7.53 (d, *J* = 3.2 Hz, 2H), 7.50 (d, *J* = 2.0 Hz, 2H), 7.26 (s, 2H), 3.96 (s, 6H), 3.92 (s, 3H); ^13^C NMR (125 MHz, CDCl_3_) δ 153.81 (2C), 146.58, 146.52, 138.47, 137.57, 136.57, 136.38, 133.42, 133.11, 132.44, 129.17 (2C), 127.29 (2C), 104.61 (2C), 60.99, 56.32 (2C); HRMS calcd for C_20_H_19_BrNO_3_ [M + H]^+^400.0548, found 400.0545.

##### 3-(4-Nitrophenyl)-5-(3,4,5-trimethoxyphenyl)pyridine (10p)

Yellow solid; yield: 68%; m.p. 175–177 °C; ^1^H NMR (500 MHz, CDCl_3_) δ 8.87 (s, 2H), 8.36 (d, *J* = 8.9 Hz, 2H), 8.02 (s, 1H), 7.81 (d, *J* = 8.9 Hz, 2H), 6.80 (s, 2H), 3.95 (s, 6H), 3.91 (s, 3H); ^13^C NMR (125 MHz, CDCl_3_) δ 153.89 (2C), 148.39, 147.75, 146.83, 144.13, 138.70, 137.28, 134.46, 132.93 (2C), 128.10 (2C), 124.39 (2C), 104.68 (2C), 61.00, 56.36 (2C); HRMS calcd for C_20_H_19_N_2_O_5_ [M + H]^+^ 367.1294, found 367.1291.

##### 3-(Thiophen-3-yl)-5-(3,4,5-trimethoxyphenyl)pyridine (10q)

Yellow solid; yield: 90%; m.p. 145–147 °C; ^1^H NMR (500 MHz, CDCl_3_) δ 8.87 (s, 1H), 8.78 (s, 1H), 8.02 (t, *J* = 1.9 Hz, 1H), 7.60 (dd, *J* = 2.9, 1.3 Hz, 1H), 7.49 (dd, *J* = 5.0, 2.9 Hz, 1H), 7.44 (dd, *J* = 5.0, 1.3 Hz, 1H), 6.78 (s, 2H), 3.94 (s, 6H), 3.91 (s, 3H); ^13^C NMR (125 MHz, CDCl_3_) δ 153.83 (2C), 145.77, 145.42, 138.56, 138.31, 133.19, 132.55, 131.86, 127.27, 125.98, 124.90, 122.06, 104.63 (2C), 60.99, 56.33 (2C); HRMS calcd for C_18_H_18_NO_3_S [M + H]^+^ 328.1007, found 328.1004.

##### 5-(3,4,5-Trimethoxyphenyl)-3,3'-bipyridine (10r)

White solid; yield: 62%; m.p. 160–161 °C; ^1^H NMR (500 MHz, CDCl_3_) δ 8.86 (dd, *J* = 10.9, 2.1 Hz, 2H), 8.74 (d, *J* = 5.7 Hz, 2H), 8.02 (t, *J* = 2.2 Hz, 1H), 7.57 (d, *J* = 4.5 Hz, 2H), 6.79 (s, 2H), 3.95 (s, 6H), 3.91 (s, 3H); ^13^C NMR (125 MHz, CDCl_3_) δ 153.88, 150.57 (2C), 148.61 (2C), 146.69, 145.14, 138.65, 137.20, 133.81, 133.00, 132.66 (2C), 121.70, 104.66 (2C), 60.99, 56.35 (2C); HRMS calcd for C_19_H_19_N_2_O_3_ [M + H]^+^ 323.1396, found 323.1395.

##### 5-(3,4,5-Trimethoxyphenyl)-3,4'-bipyridine (10s)

White solid; yield: 80%; m.p. 94–95 °C; ^1^H NMR (500 MHz, CDCl_3_) δ 8.91 (s, 1H), 8.84 (s, 1H), 8.80 (s, 1H), 8.69 (d, *J* = 4.0 Hz, 1H), 7.99 (s, 1H), 7.94 (d, *J* = 7.9 Hz, 1H), 7.44 (dd, *J* = 7.8, 4.9 Hz, 1H), 6.80 (s, 2H), 3.94 (s, 6H), 3.91 (s, 3H); ^13^C NMR (125 MHz, CDCl_3_) δ 153.85 (2C), 149.47, 148.30, 147.77, 146.77, 138.58, 137.11, 134.55, 133.47, 133.43, 133.11, 132.78, 123.81, 104.62 (2C), 60.99, 56.33 (2C); HRMS calcd for C_19_H_19_N_2_O_3_ [M + H]^+^ 323.1396, found 323.1393.

##### 4-(5-(3,4,5-Trimethoxyphenyl)pyridin-3-yl)-1H-indole (10t)

White solid; yield: 51%; m.p. 209–210 °C; ^1^H NMR (500 MHz, CDCl_3_) δ 8.95 (s, 1H), 8.83 (d, *J* = 7.5 Hz, 2H), 8.17 (s, 1H), 7.50 (d, *J* = 8.1 Hz, 1H), 7.37–7.30 (m, 2H), 7.29–7.23 (m, 1H), 6.85 (s, 2H), 6.73 (s, 1H), 3.95 (s, 6H), 3.93 (s, 3H); ^13^C NMR (125 MHz, CDCl_3_) δ 153.77 (2C), 148.16, 146.33, 138.28, 136.92, 136.76, 136.34, 134.40, 133.75, 130.34, 126.19, 125.29, 122.37, 119.98, 111.35, 104.60 (2C), 101.37, 61.00, 56.29 (2C); HRMS calcd for C_22_H_21_N_2_O_3_ [M + H]^+^ 361.1552, found 361.1550.

##### 3-(Naphthalen-2-yl)-5-(3,4,5-trimethoxyphenyl)pyridine (10u)

White solid; yield: 76%; m.p. 145–147 °C; ^1^H NMR (500 MHz, CDCl_3_) δ 8.95 (s, 1H), 8.82 (s, 1H), 8.11 (d, *J* = 7.5 Hz, 2H), 7.99 (d, *J* = 8.5 Hz, 1H), 7.94 (d, *J* = 9.0 Hz, 1H), 7.90 (d, *J* = 9.0 Hz, 1H), 7.77 (d, *J* = 10.2 Hz, 1H), 7.58–7.50 (m, 2H), 6.84 (s, 2H), 3.96 (s, 6H), 3.93 (s, 3H); ^13^C NMR (125 MHz, CDCl_3_) δ 153.81 (2C), 147.21, 146.95, 138.44, 136.95, 136.60, 134.98, 133.61, 133.57, 133.05, 132.95, 128.97, 128.22, 127.74, 126.70, 126.56, 126.33, 125.07, 104.68 (2C), 61.00, 56.35 (2C); HRMS calcd for C_24_H_22_NO_3_ [M + H]^+^ 372.1600, found 372.1598.

### Biology experiment

#### MTT assay

The *in vitro* antiproliferative activity of the target compounds and CA-4 was measured by a standard MTT (meilunbio®, China) assay. Cervical cancer (HeLa), gastric adenocarcinoma (SGC-7901) and breast cancer (MCF-7) were used, respectively. Cells were inoculated in 96-well plates at a density of 2 × 10^3^/well. After 24 h, the target concentration of drug was added to each well and incubated for 72 h at 37 °C under 5% CO_2_. 20 μL of fresh medium containing 5 mg/ml MTT solution was added and incubation continued for 4 h. After removing the medium containing MTT from each well, 150 μL of dimethyl sulfoxide (DMSO) was added to each well until the purple formazan crystals was completely dissolved, placed in a multimode plate reader Victor Nivo 3S (Perkinelmer, USA) and the absorbance was measured at 490 nm.^26^

#### Tubulin polymerisation assay

The microtubule polymerisation ability of 10 and 15 μM **10t** was tested *in vitro* using the Microtubulin Kit (Cytoskeleton-Cat. #BK011P) by suspending microtubulin in ice-cold G-PEM buffer (80 mM PIPES, 2 mM MgCl_2_, 0.5 mM EGTA, 1 mM GTP, 20% (v/v) glycerol) and adding to 96-well plates provided by the kit. **10t** were compared with the positive control drug 5 μM CA-4 and the negative control drug 5 μM taxol. The polymerisation of microtubule proteins was monitored at 1 min intervals (emission wavelength: 450 nm, excitation wavelength: 360 nm) for 61 min at 37 °C using a microplate reader (Tecan, Austria) and the absorbance values were used for calculation.^11^^,^^27^

#### Immunofluorescence assay

HeLa cells were inoculated in 6-well plates at a density of 2 × 10^5^ per well and grown for 24 h. Cells were treated with 2-fold IC_50_ of positive drugs CA-4 or the 2-fold IC_50_ target compound for 24 h. Control and treated cells were washed in PBS, fixed in 4% paraformaldehyde solution for 20 min, then washed three times in PBST and permeabilised with 0.5% (v/v) Triton X-100 in PBS for 10 min. Cells were then closed with 3% bovine serum albumin (BSA) for 60 min. The *α*-microtubulin antibody (Abclonal, China) was diluted with 3% BSA (1:100) and incubated for 3 h. Cells were washed three times with PBST for 10 min each time to remove unbound primary antibody, then FITC-conjugated antimouse secondary antibody (1:100) and DAPI (1:100) were diluted with 3% BSA and incubated for a further 1 h. Cells were washed three times with PBST for 10 min each time to remove unbound secondary antibody and DAPI, and then immunofluorescence was detected by fluorescence confocal microscopy (Nikon, Japan).^28^^,^^29^

#### Cell cycle analysis

HeLa cells were inoculated in 6-well plates at a density of 2 × 10^5^ per well and grown for 24 h. Cells were treated with 1-fold IC_50_, 2-fold IC_50_ and 3-fold IC_50_ **10t** for 24 h. Cells were collected by centrifugation, washed with PBS and fixed overnight in ice-cold 70% ethanol. The fixed cells were collected by centrifugation and 100 μL of ribonuclease (RNase) was added. After a water bath at 37 °C for 30 min, 400 μL PI staining was added and the samples were stained for 30 min in the dark at 4 °C. Finally, the samples were analysed by flow cytometry (Beckman Coulter, USA). The data were processed and evaluated using software.^30^^,^^31^

#### Cell apoptosis analysis

HeLa cells were inoculated in 6-well plates at a density of 2 × 10^5^ per well and grown for 24 h. Cells were treated with 1-fold IC_50_, 2-fold IC_50_ and 3-fold IC_50_ **10t** for 48 h. After collecting the cells with trypsin without EDTA, the cells were washed twice with pre-cooled PBS, and Binding Buffer was added to the cell pellet to resuspend the cells. The concentration reached 1 × 10^6^/ml, then stained with 5 μL Annexin-V FITC and 5 μL PI for 15 min in the dark, and measured by flow cytometry (Beckman Coulter, USA).^32^^,^^33^

#### Molecular modelling

Molecular modelling studies were performed using the CDOCKER program of the Discovery Studio 3.0 software, and the crystal structure of the tubulin complex (PDB: 1SA0) was retrieved from the RCSB protein database (http://www.rcsb.org/pdb). Hydrogen atoms are added to the crystal after the ligand is extracted. Charges are added to biopolymers via the CHARMm force field. Finally, **10t** was docked to the colchicine site of tubulin using the CDOCKER protocol.^34^

## Supplementary Material

Supplemental Material
