# Supplementary material for: Design, synthesis and biological evaluation of novel diarylpyridine derivatives as tubulin polymerisation inhibitors
Source: J Enzyme Inhib Med Chem. 2022 Oct 5;37(1):2755–64. doi: 10.1080/14756366.2022.2130284 (PMC9553186; doi:10.1080/14756366.2022.2130284)
Supplement: Supplemental Material [file IENZ_A_2130284_SM1002.pdf]

Supplementary data

Design, synthesis and biological evaluation of novel diarylpyridine derivatives as tubulin polymerization inhibitors

Shanbo Yang [a,b#], Chao Wang [b#*], Lingyu Shi [a,b], Jing Chang [a,b], Yujing Zhang [c], Jingsen Meng [a,b], Wenjing Liu [a,b], Jun Zeng [a,b], Renshuai Zhang [b], Yingchun Shao [b*], Dongming Xing [b,d*]

[a] School of Basic Medicine of Qingdao University, Qingdao, 266071, Shandong, China

[b] Cancer Institute, The Affiliated Hospital of Qingdao University, Qingdao, 266071, Shandong China

[c] The Affiliated Cardiovascular Hospital of Qingdao University, Qingdao University, Qingdao, 266071, Shandong, China

[d] School of Life Sciences, Tsinghua University, Beijing, 100084, China

[#] Share the first author

E-mail addresses: wangchao20086925@126.com (C. Wang), 14268657@qq.com (Y. Shao), xdm_tsinghua@163.com (D. Xing).

Information Classification: General

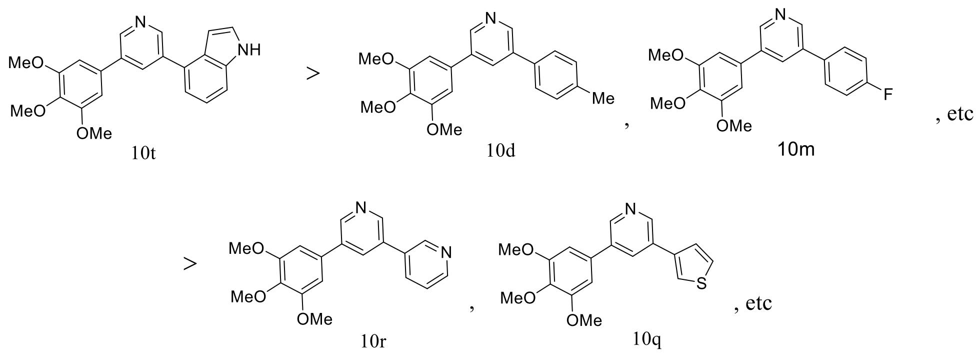

Figure 1. The SAR of the synthesized compounds.

Contents: ^{1}H-NMR and ^{13}C-NMR spectra of all target compounds

*3-phenyl-5-(3,4,5-trimethoxyphenyl)pyridine (**10a**)*

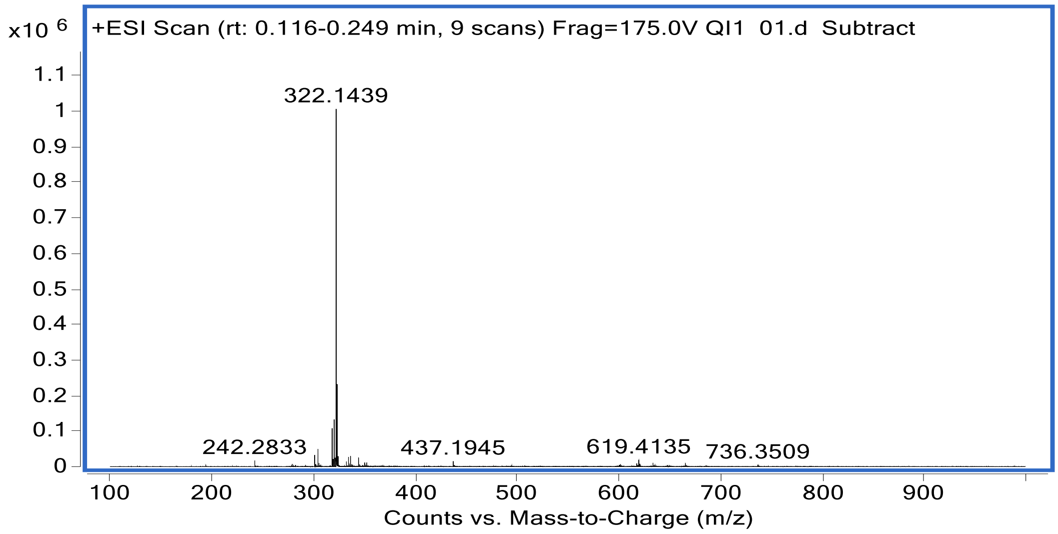

Information Classification: General

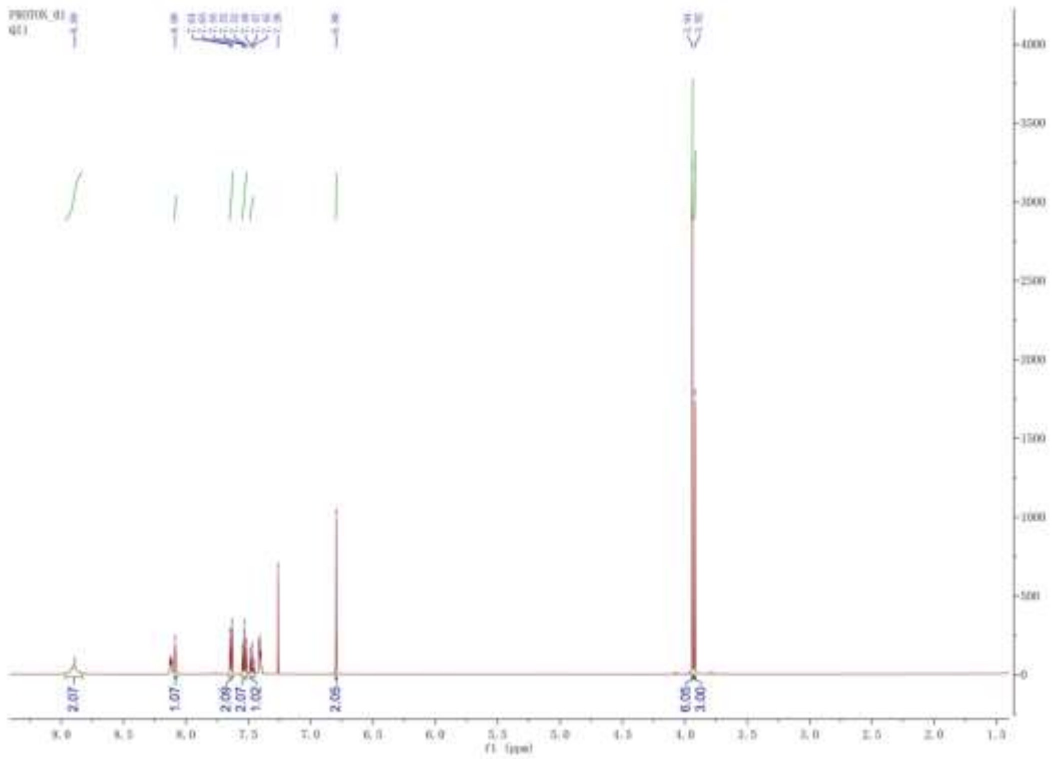

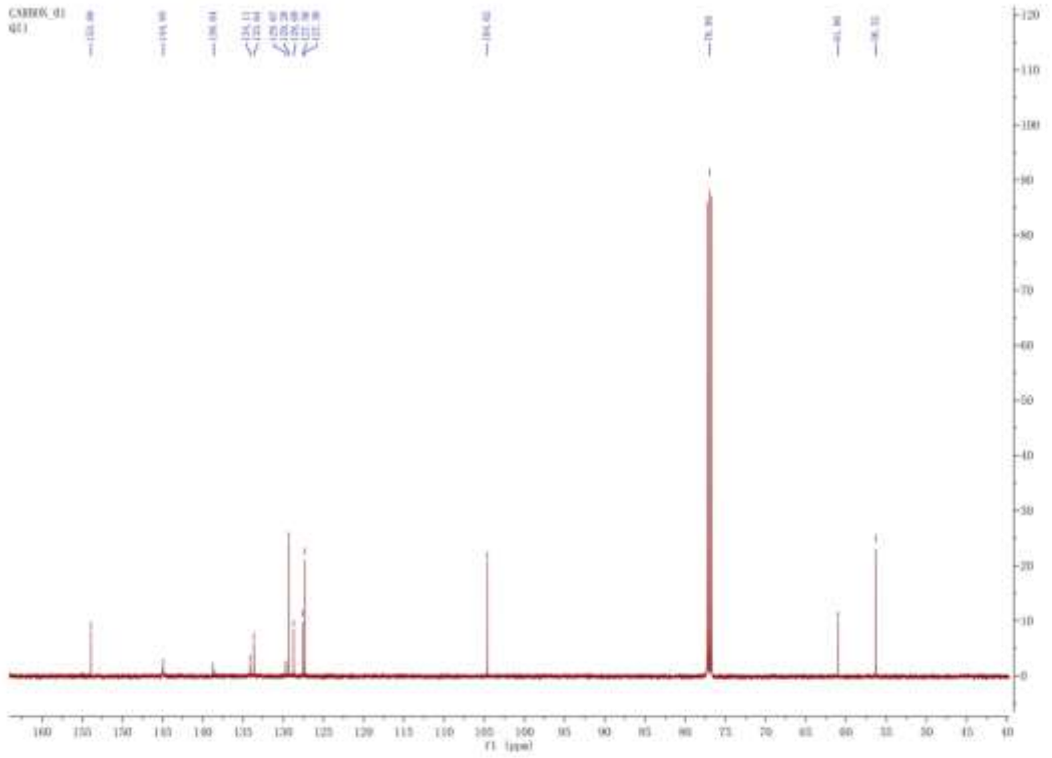

*3-(o-tolyl)-5-(3,4,5-trimethoxyphenyl)pyridine (**10b**)*

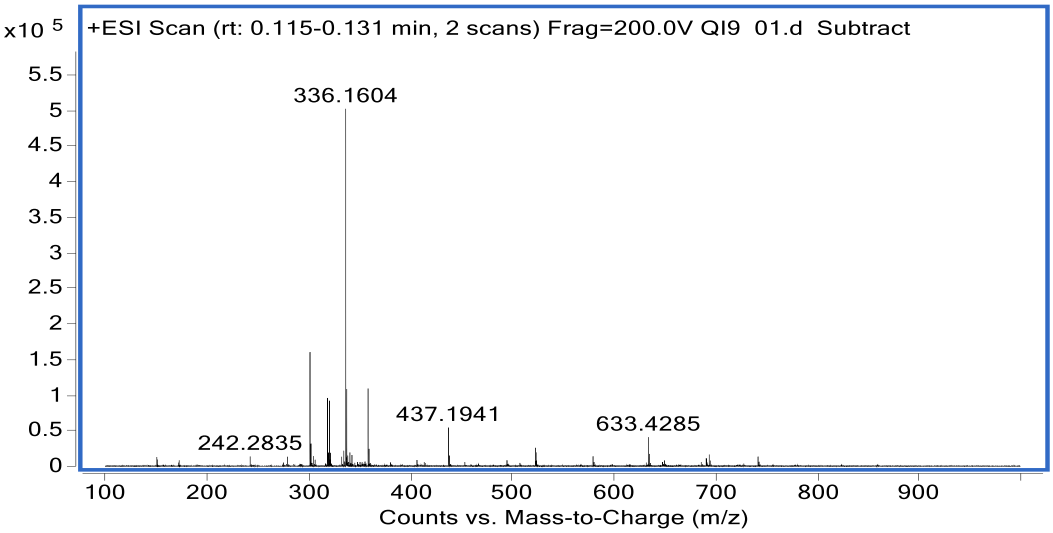

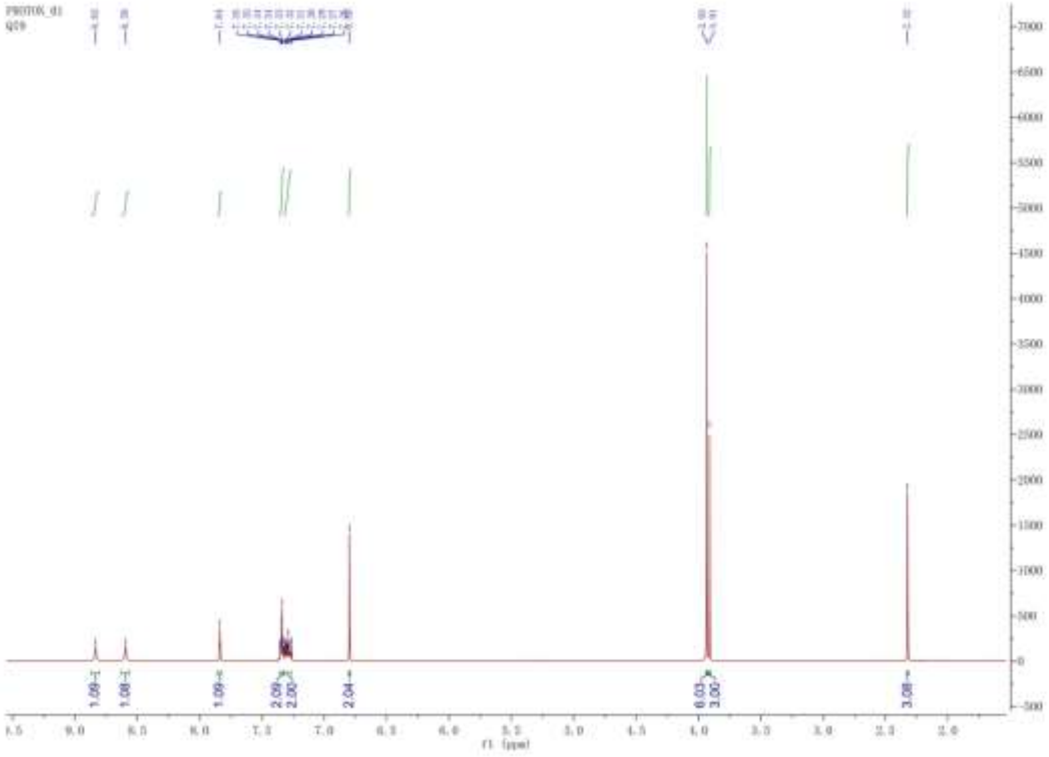

Information Classification: General

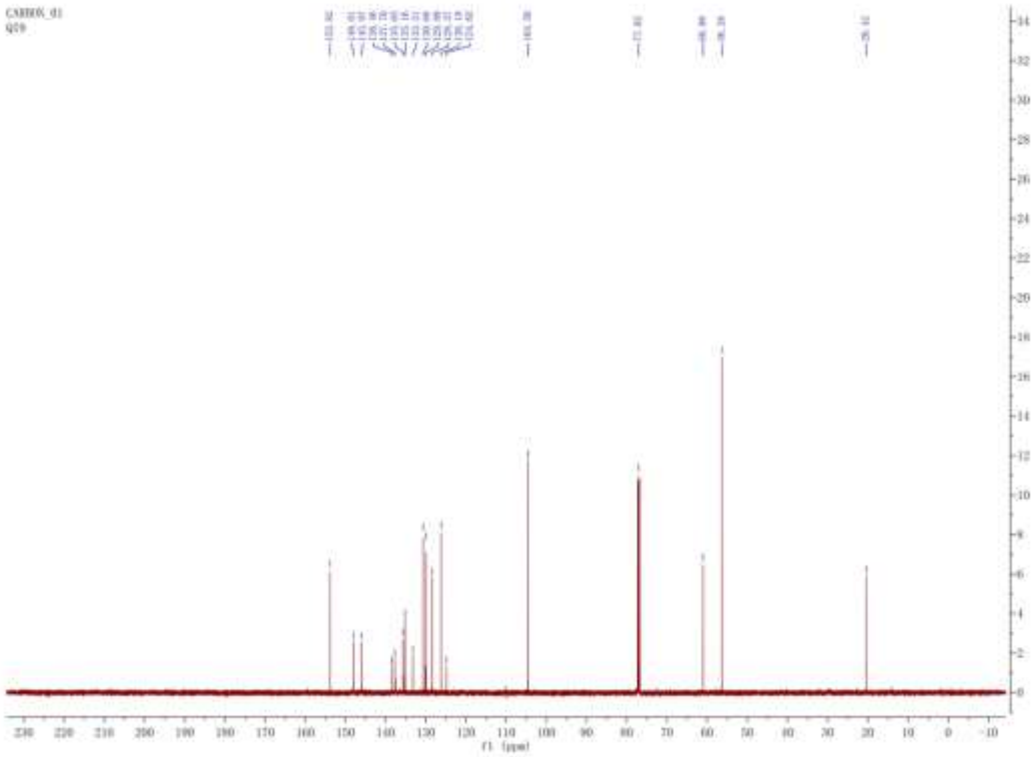
CARBON_01
Q19
f1 (ppm)

*3-(m-tolyl)-5-(3,4,5-trimethoxyphenyl)pyridine (**10c**)*

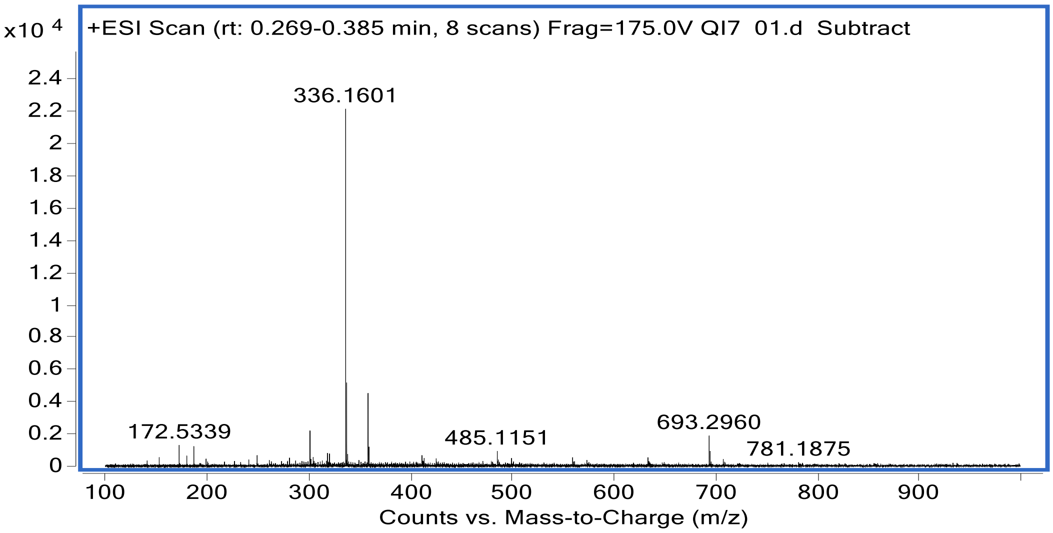

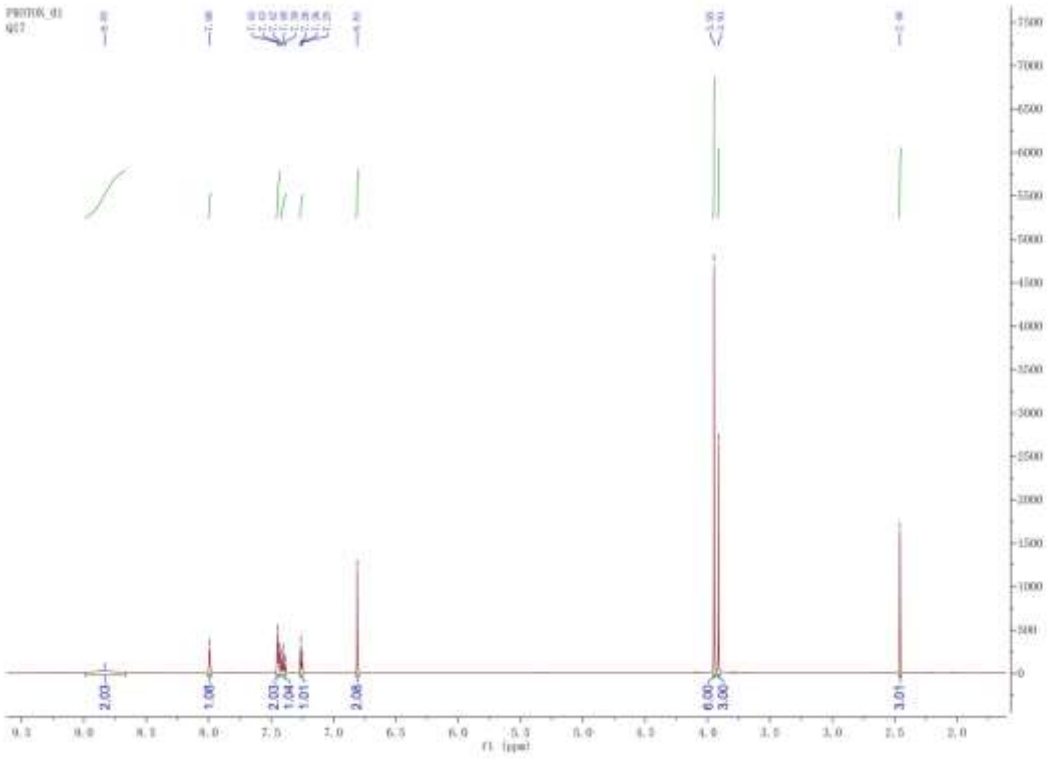

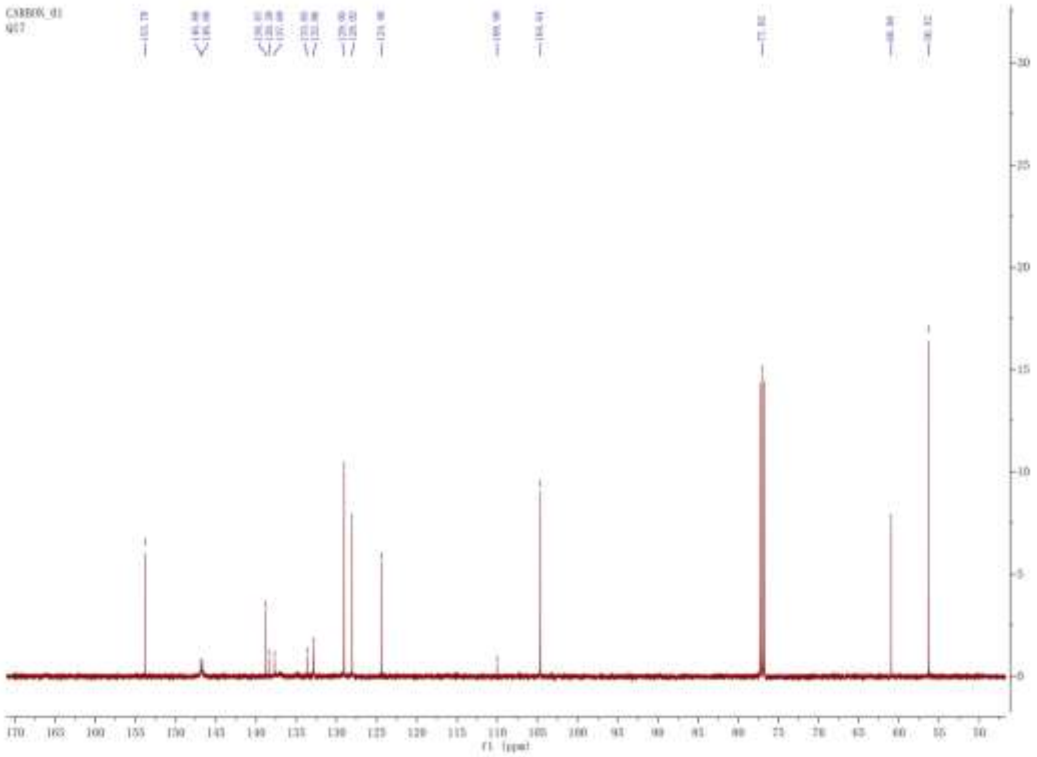

Information Classification: General

*3-(p-tolyl)-5-(3,4,5-trimethoxyphenyl)pyridine (**10d**)*

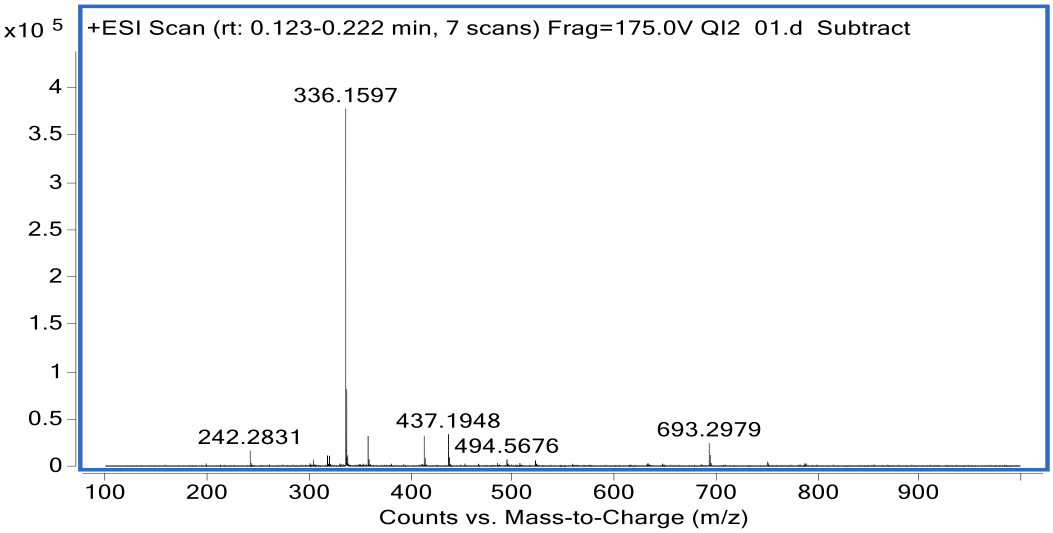

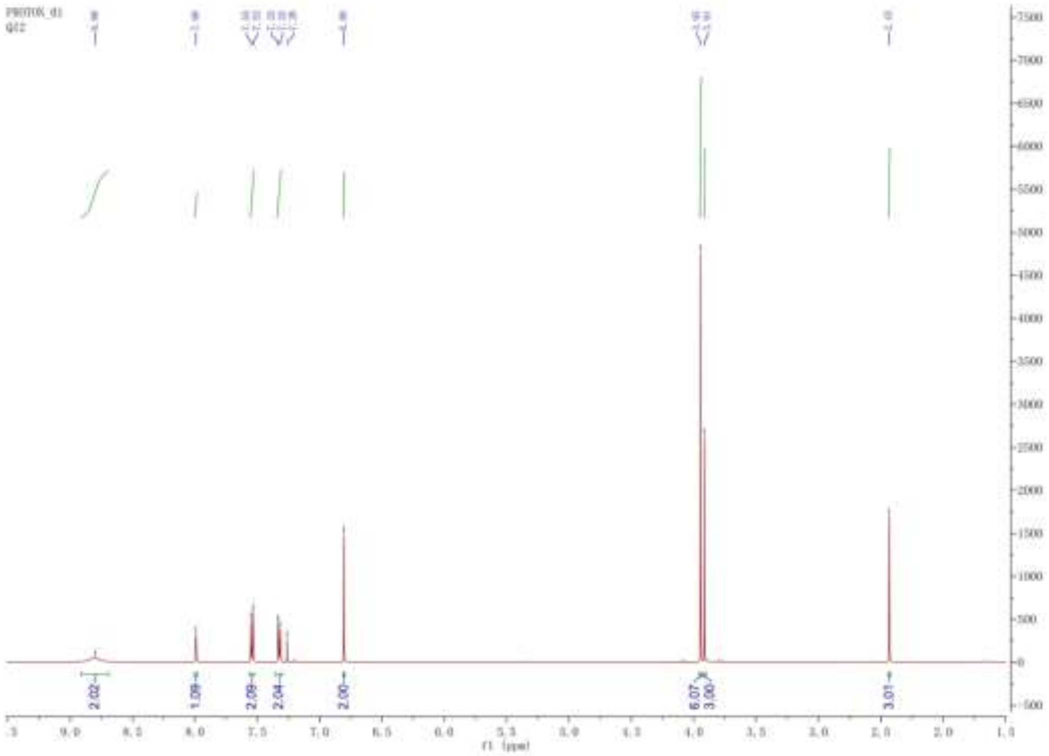

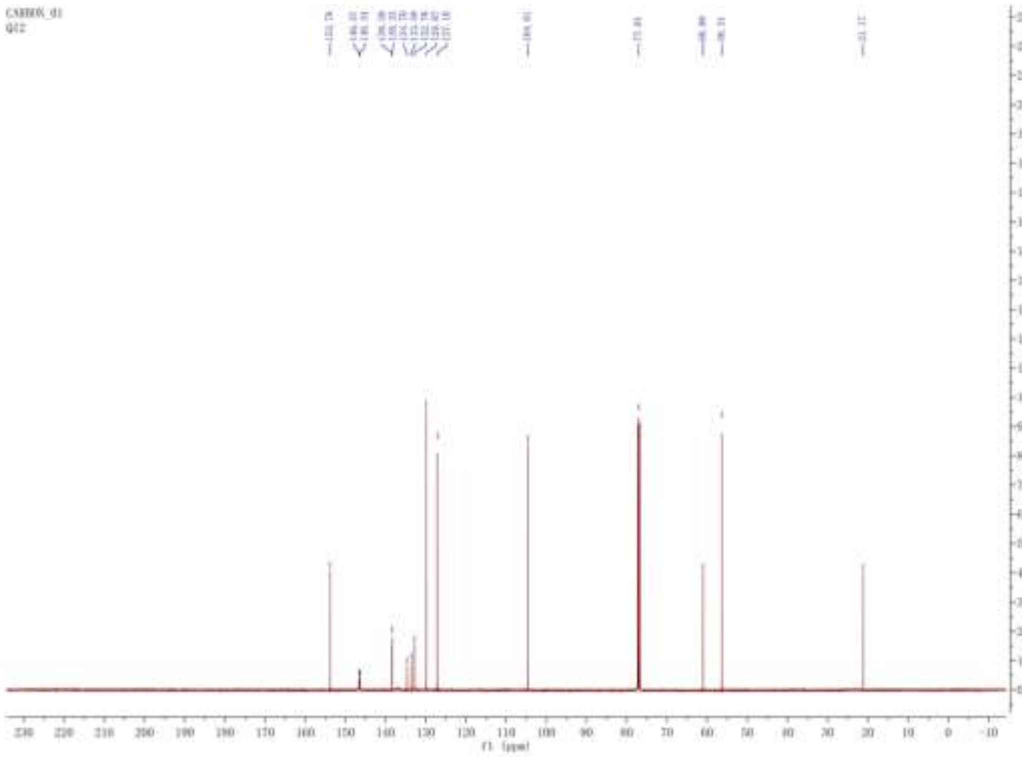

*3-(3,4-dimethylphenyl)-5-(3,4,5-trimethoxyphenyl)pyridine (**10e**)*

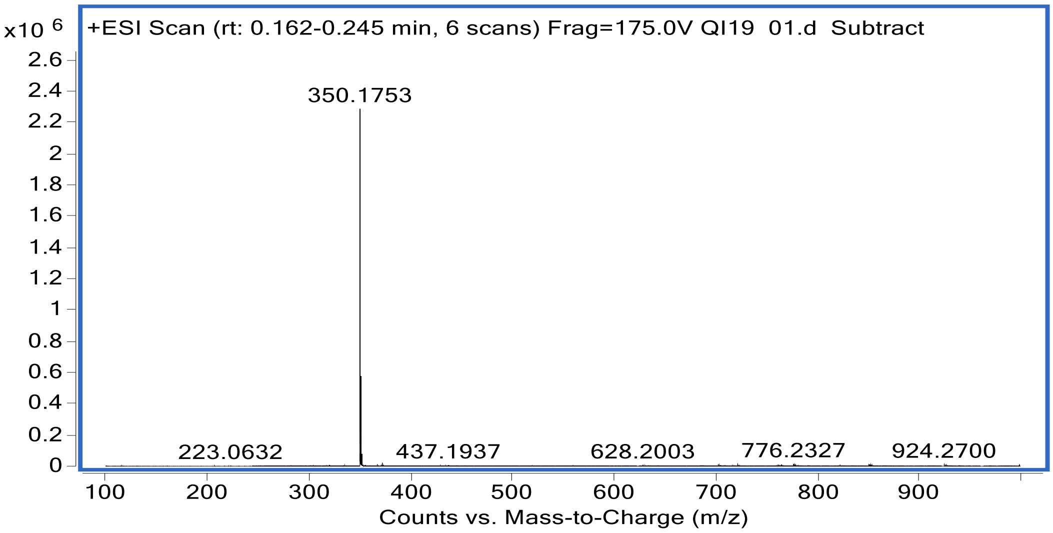

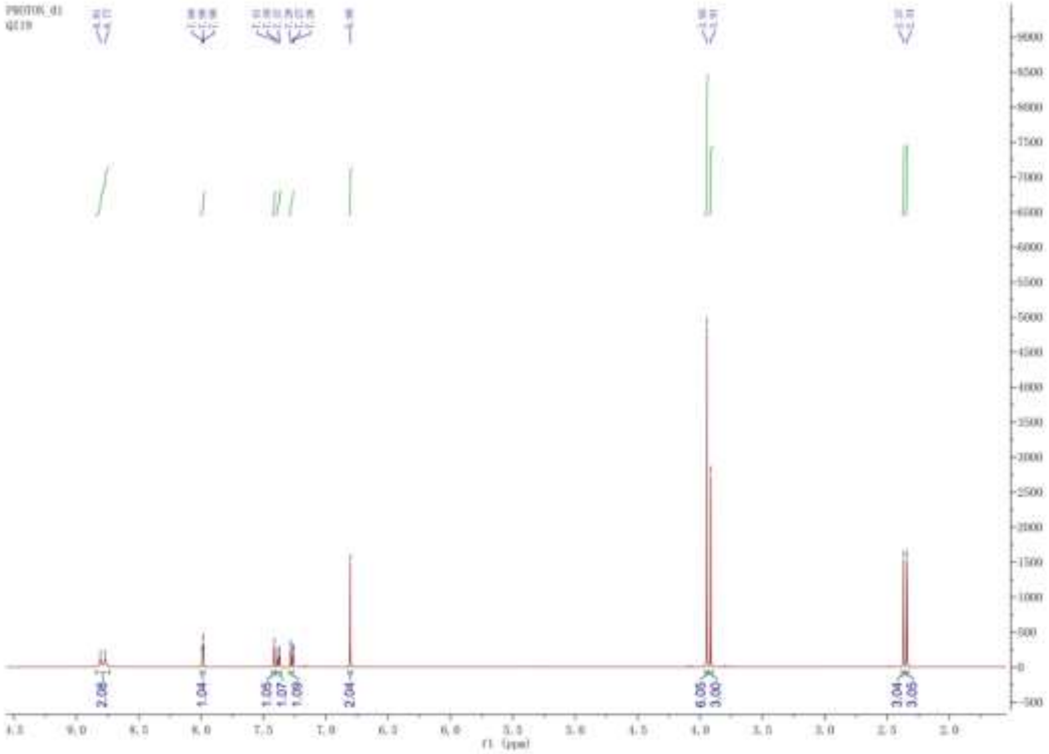

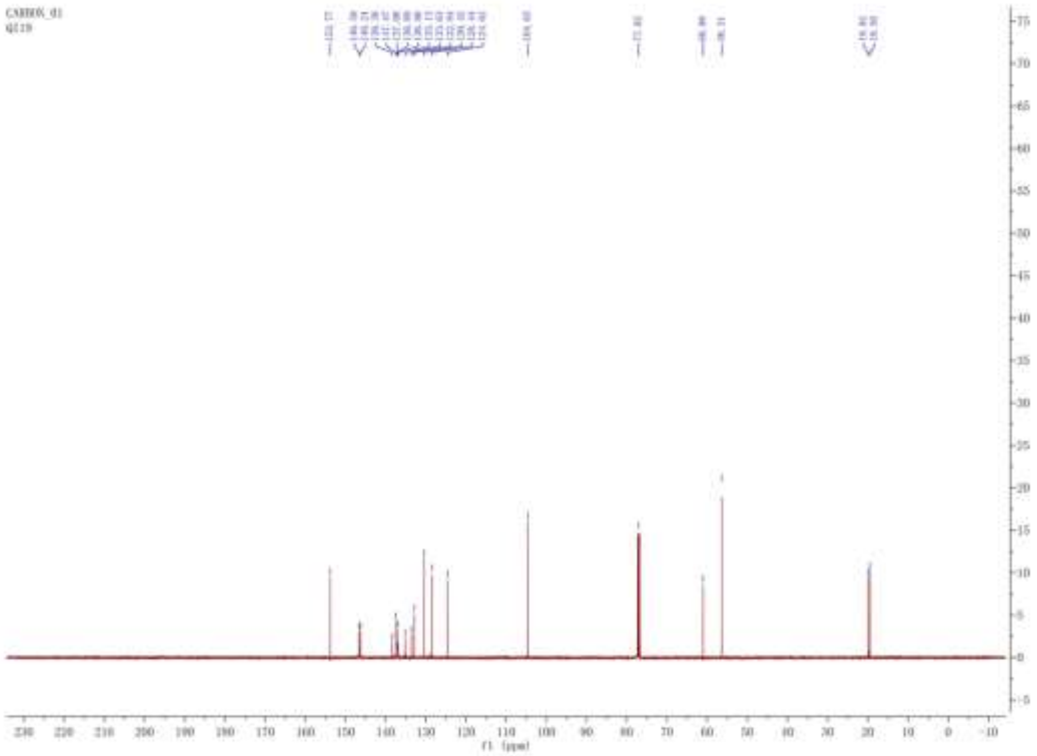

*3-(2-methoxyphenyl)-5-(3,4,5-trimethoxyphenyl)pyridine (**10f**)*

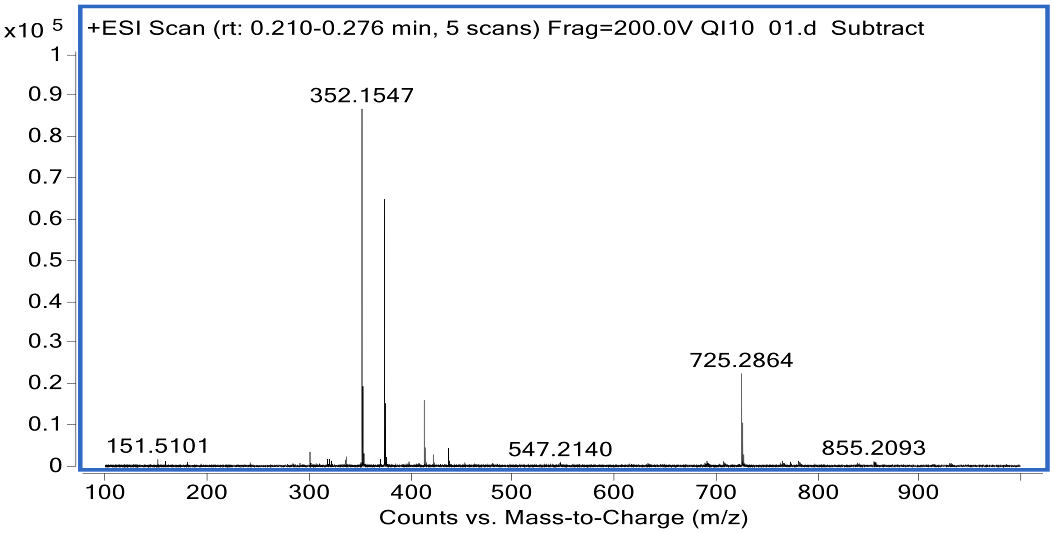

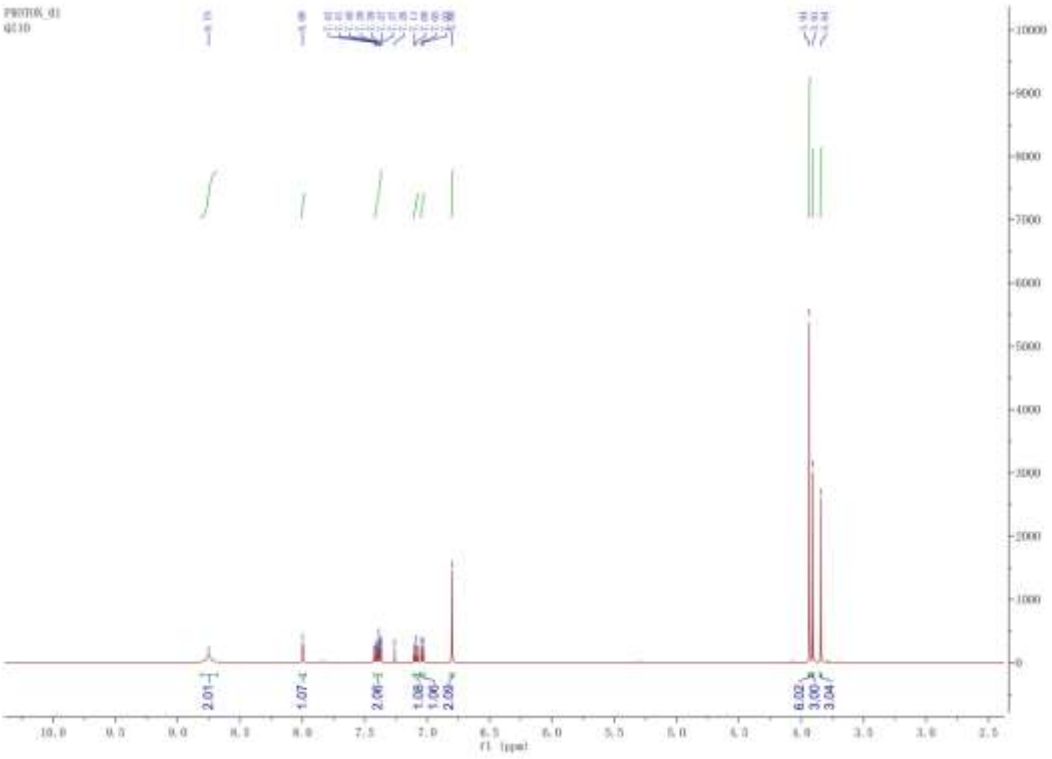

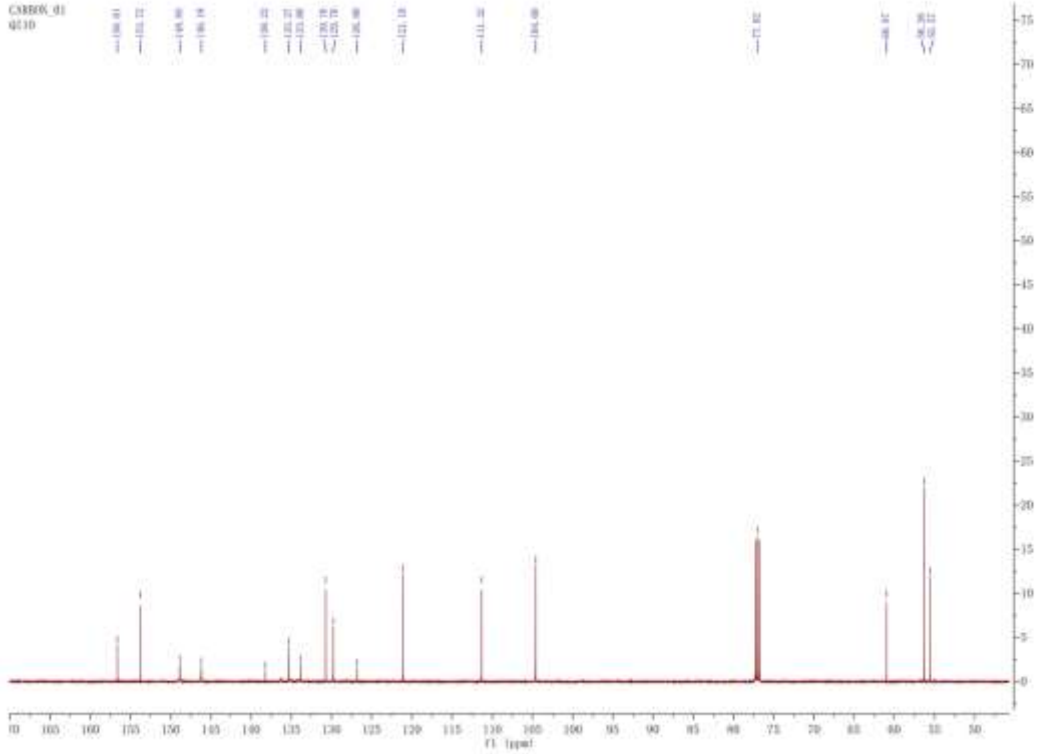

Information Classification: General

*3-(3-methoxyphenyl)-5-(3,4,5-trimethoxyphenyl)pyridine (**10g**)*

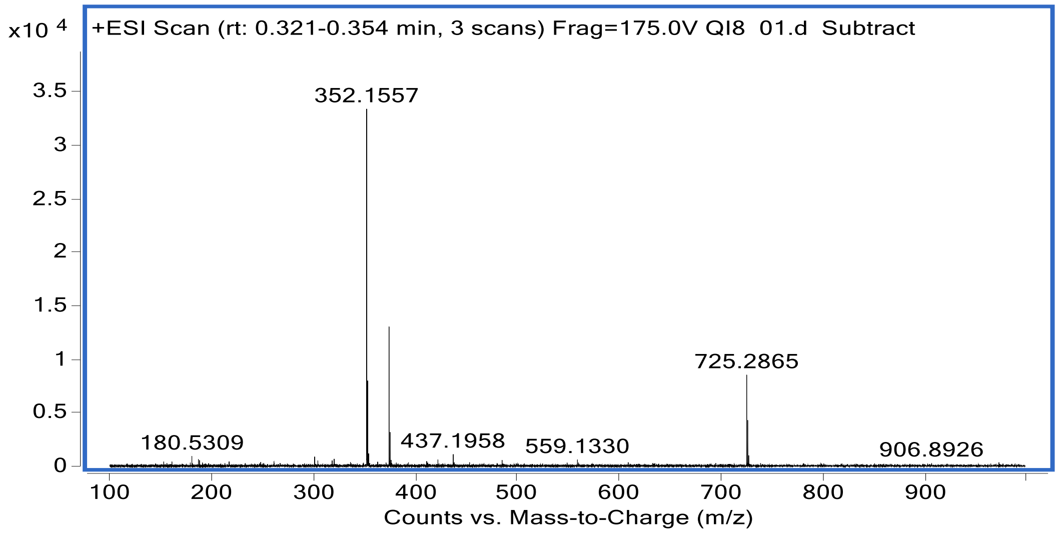

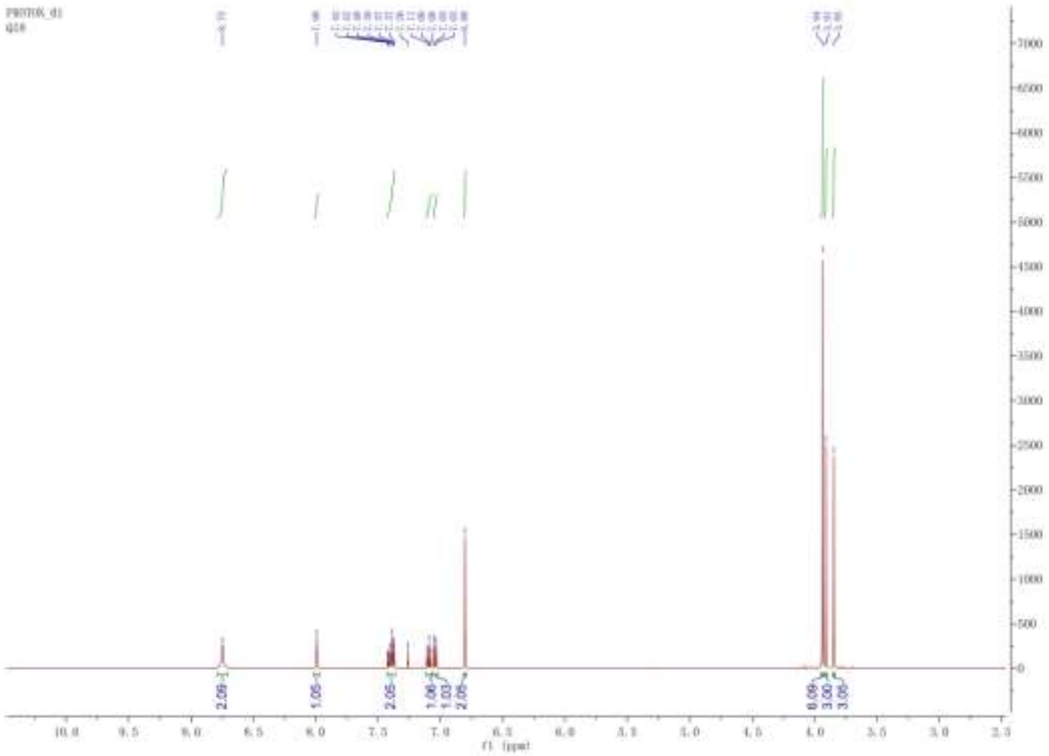

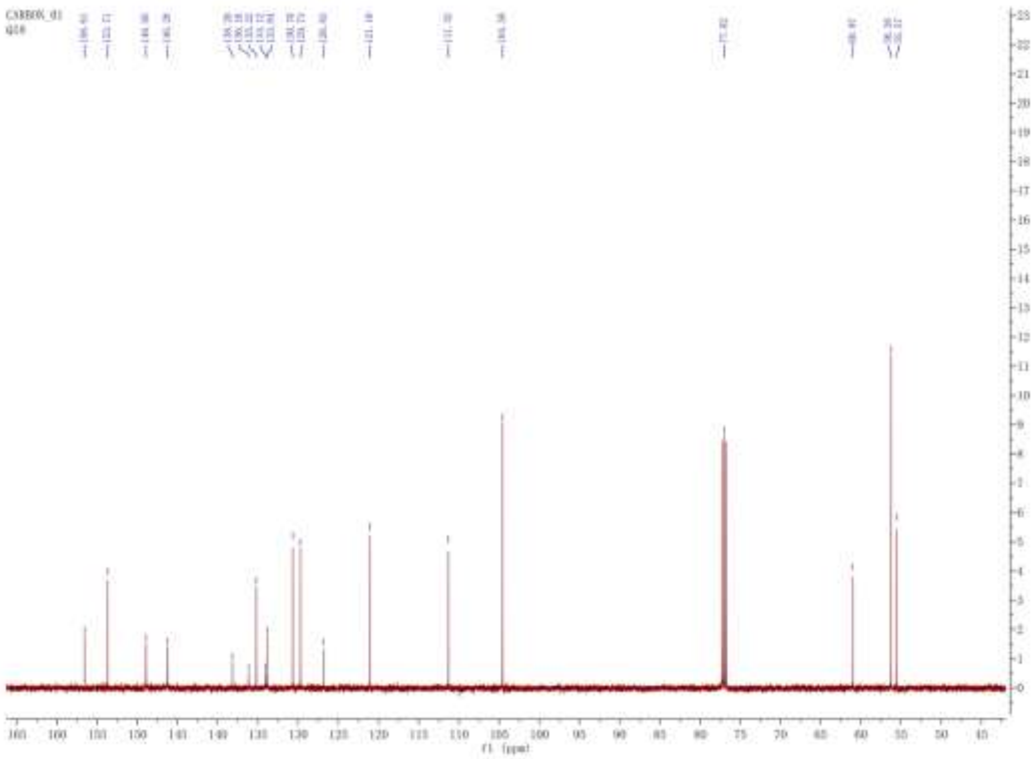
CARBON_01
f1 (ppm)

*3-(4-methoxyphenyl)-5-(3,4,5-trimethoxyphenyl)pyridine (**10h**)*

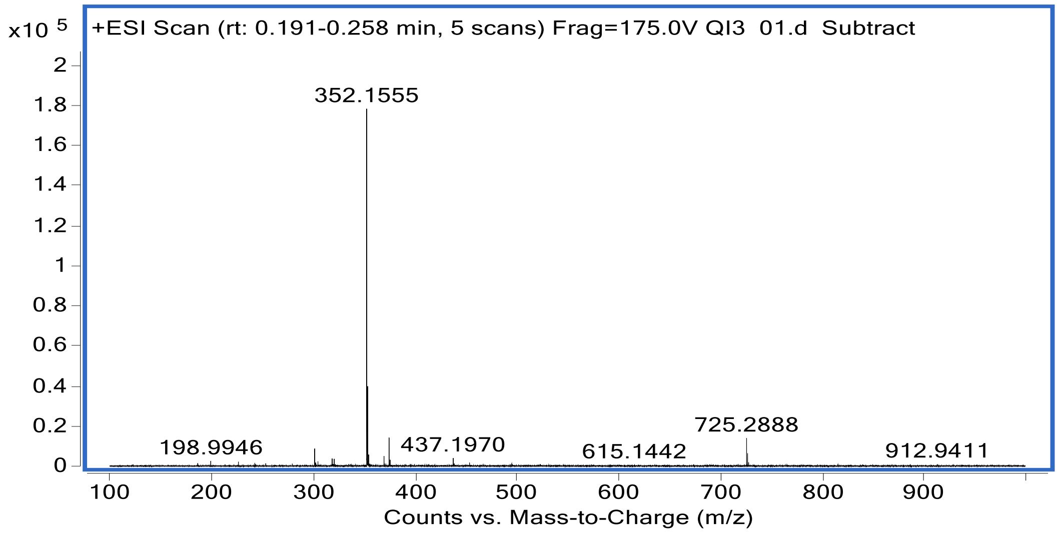

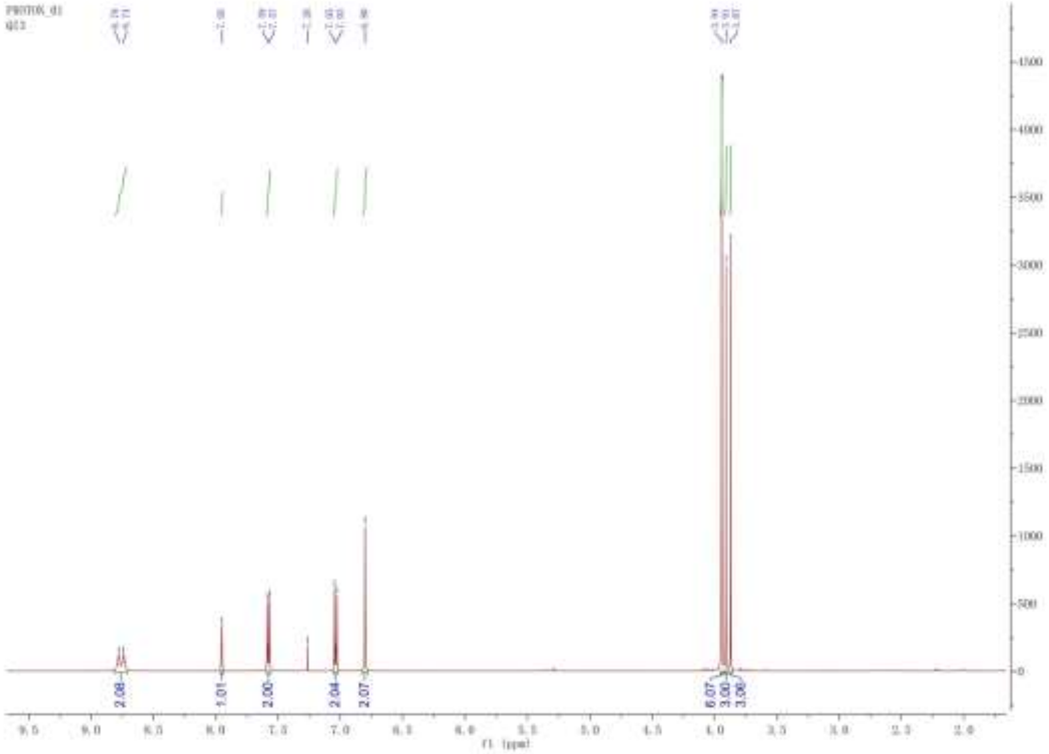

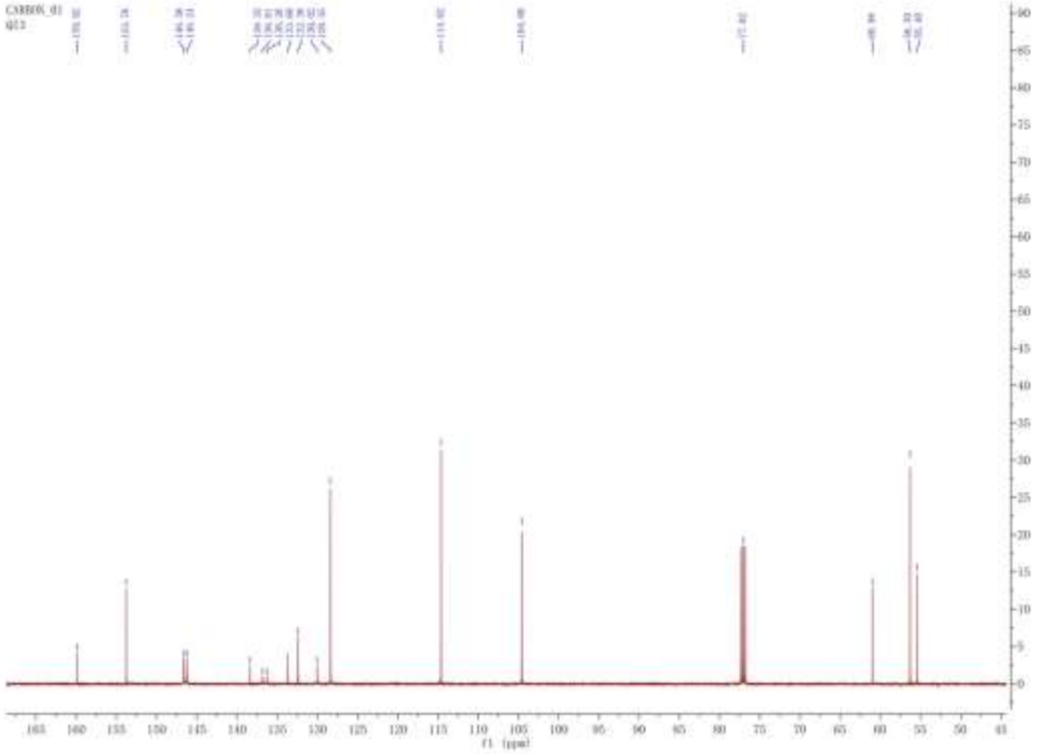

*2-methoxy-5-(5-(3,4,5-trimethoxyphenyl)pyridin-3-yl)phenol (**10i**)*

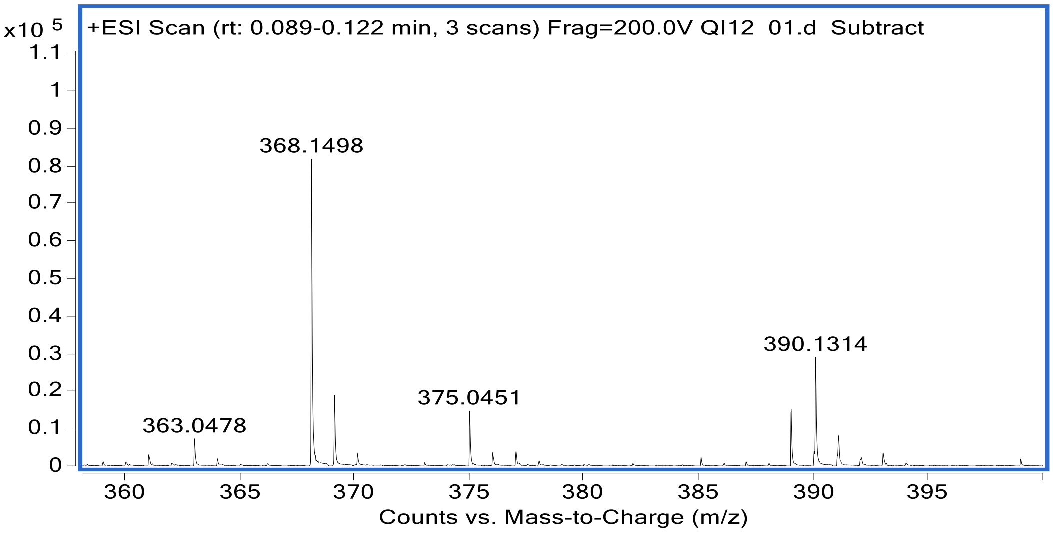

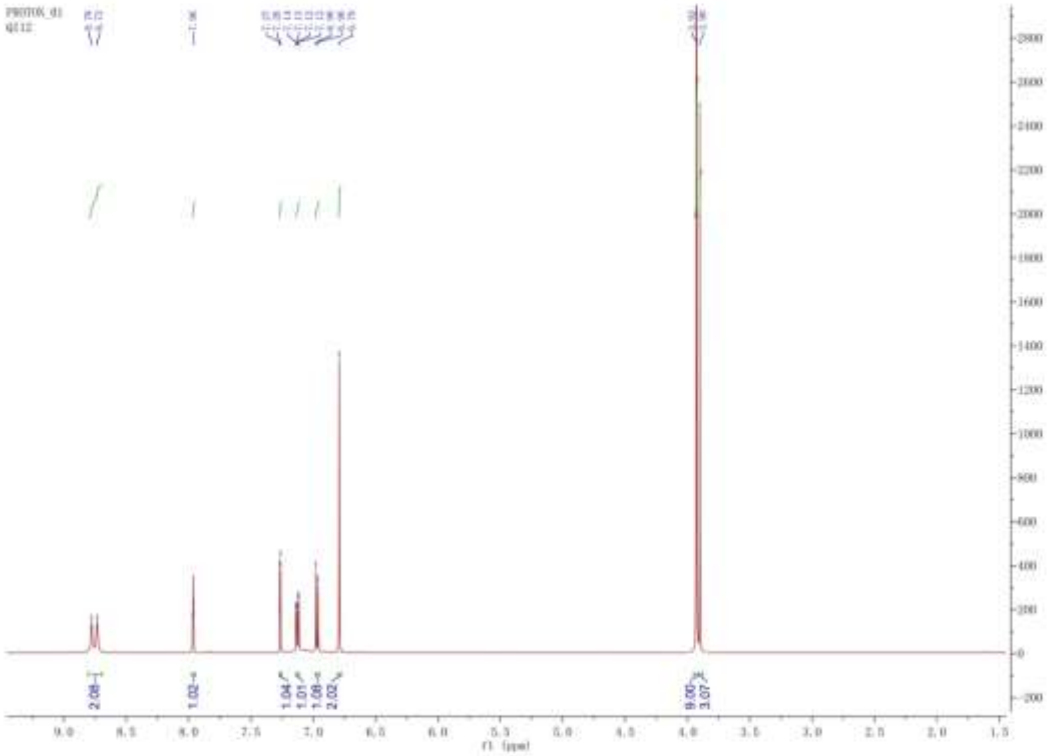

Information Classification: General

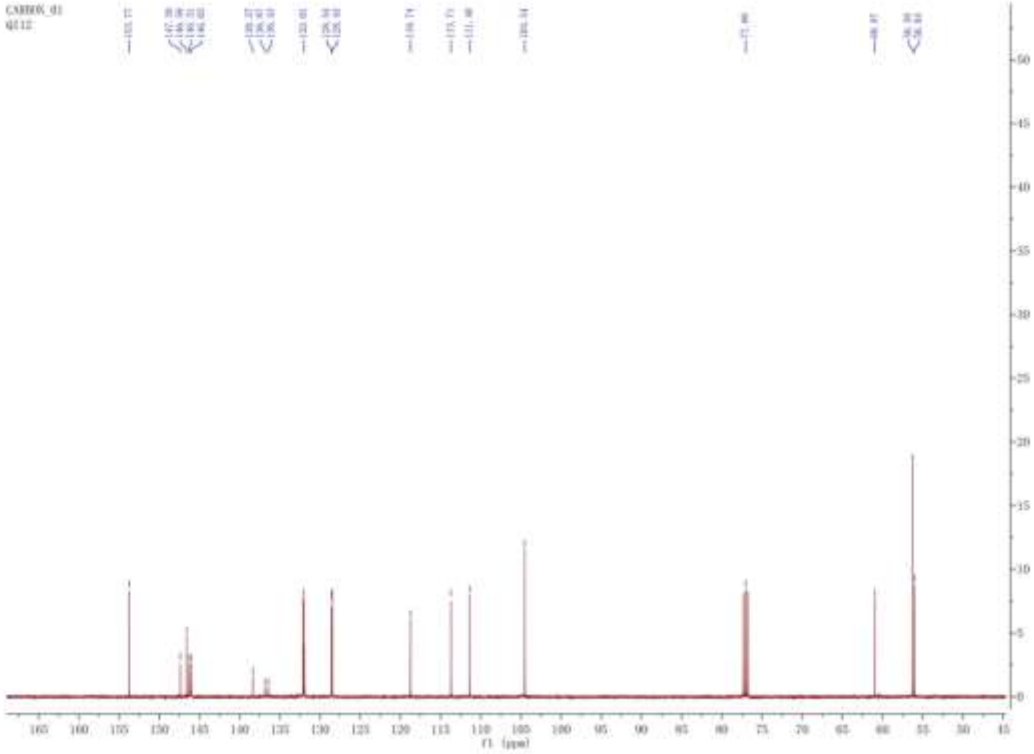
CARBON_01
f1 (ppm)

*3-(3,4-dimethoxyphenyl)-5-(3,4,5-trimethoxyphenyl)pyridine (**10j**)*

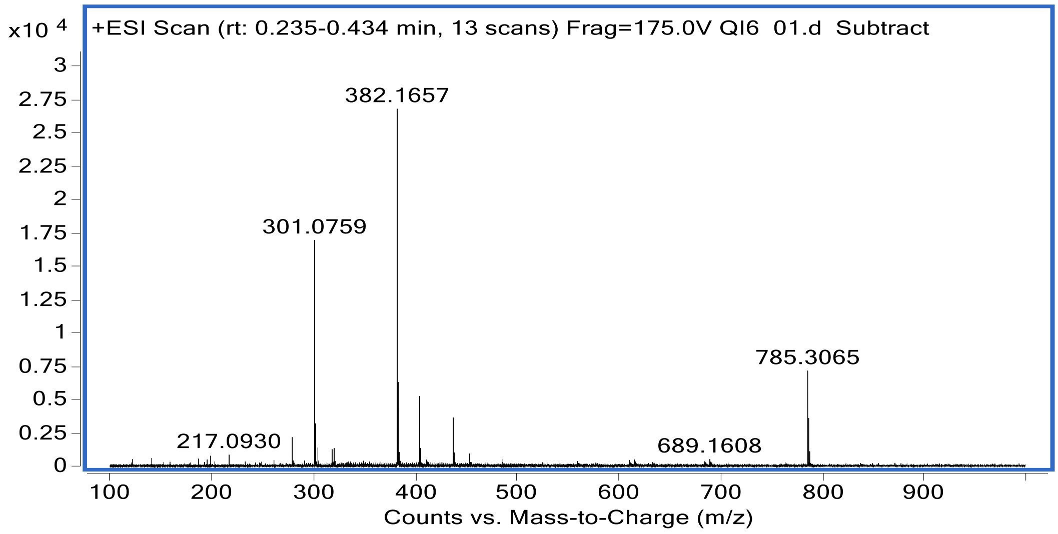

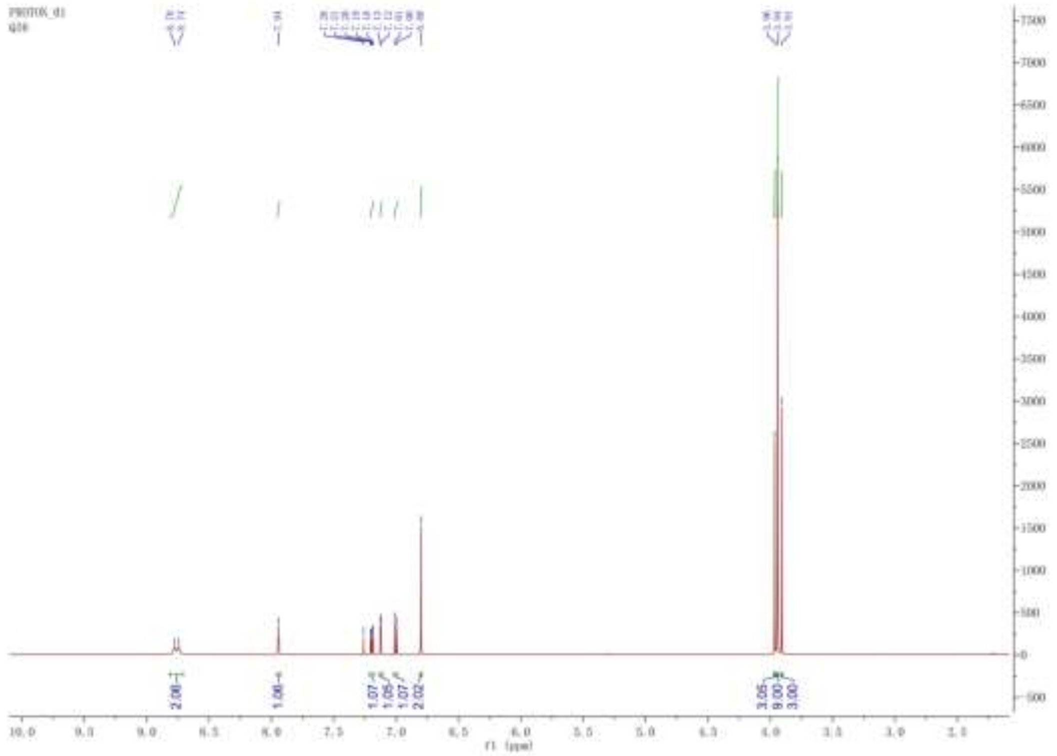

Information Classification: General

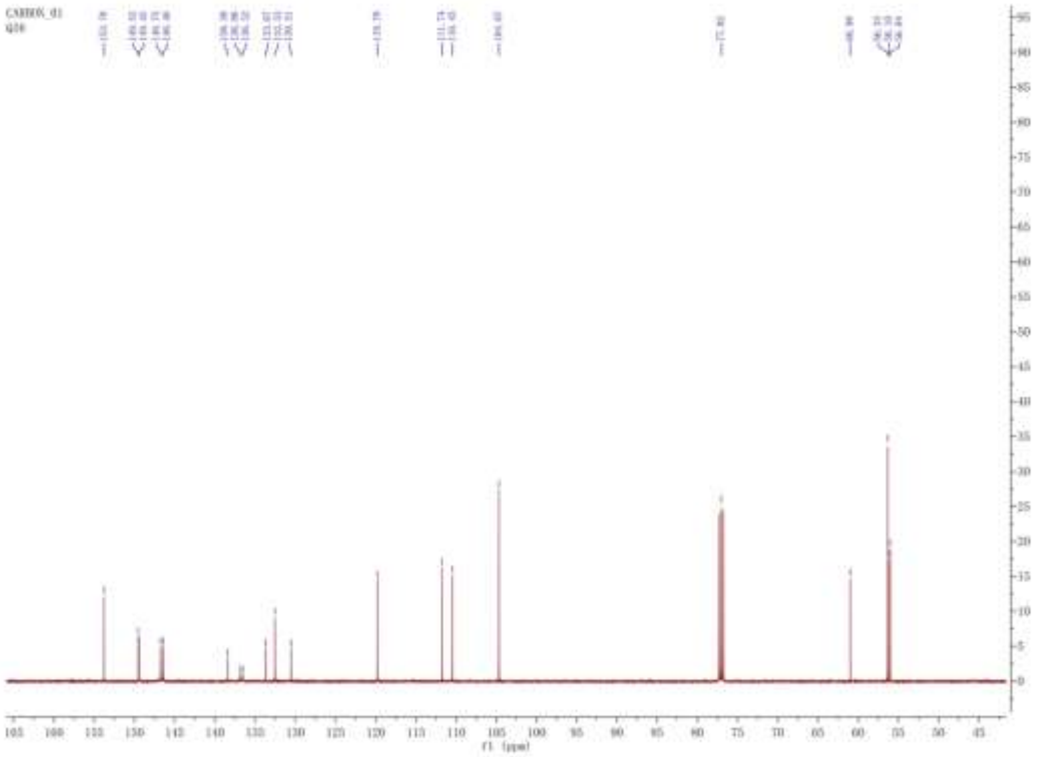

Information Classification: General

*3-(4-ethoxyphenyl)-5-(3,4,5-trimethoxyphenyl)pyridine (**10k**)*

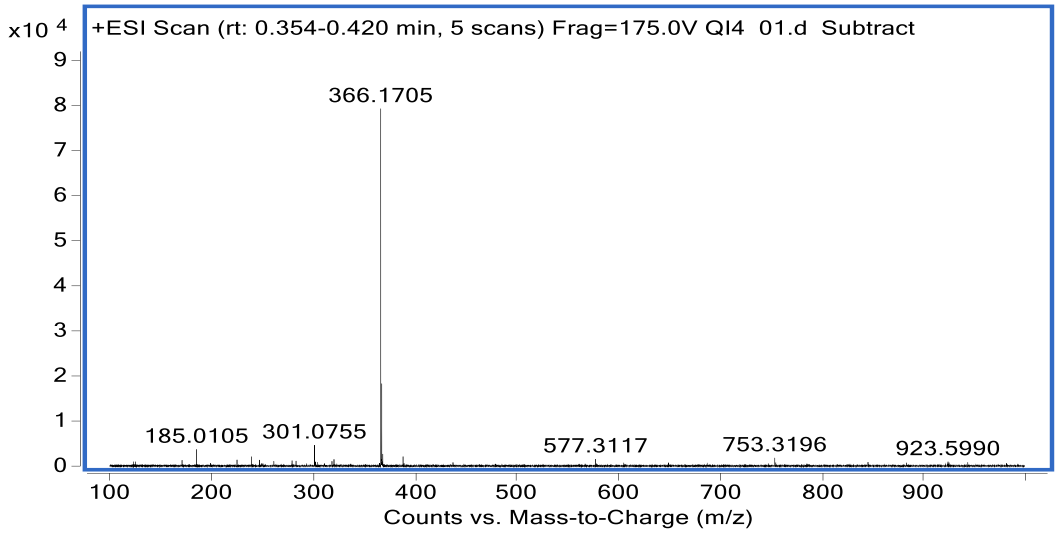

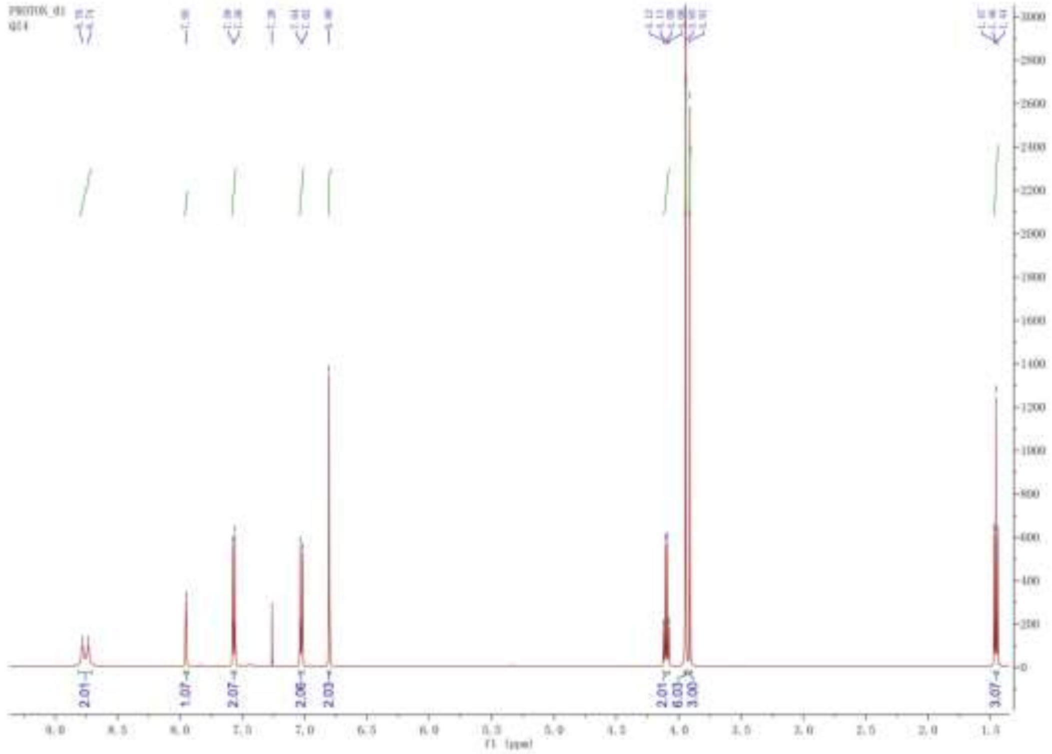

Information Classification: General

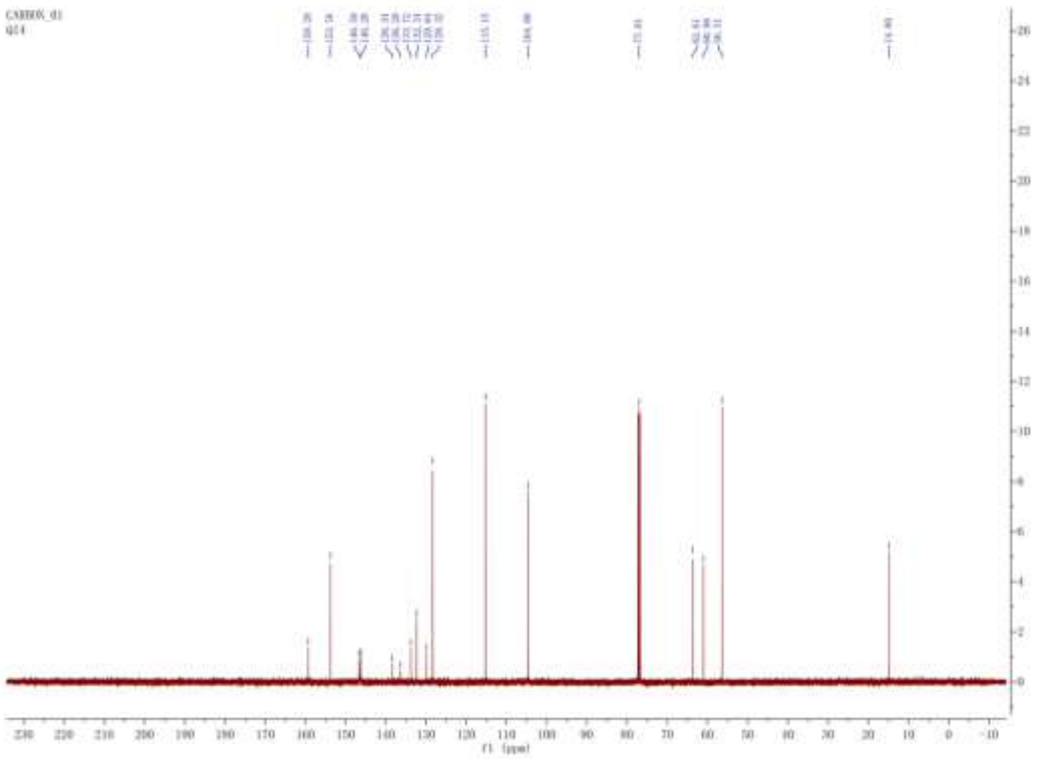

Information Classification: General

*4-(5-(3,4,5-trimethoxyphenyl)pyridin-3-yl)phenol (**10l**)*

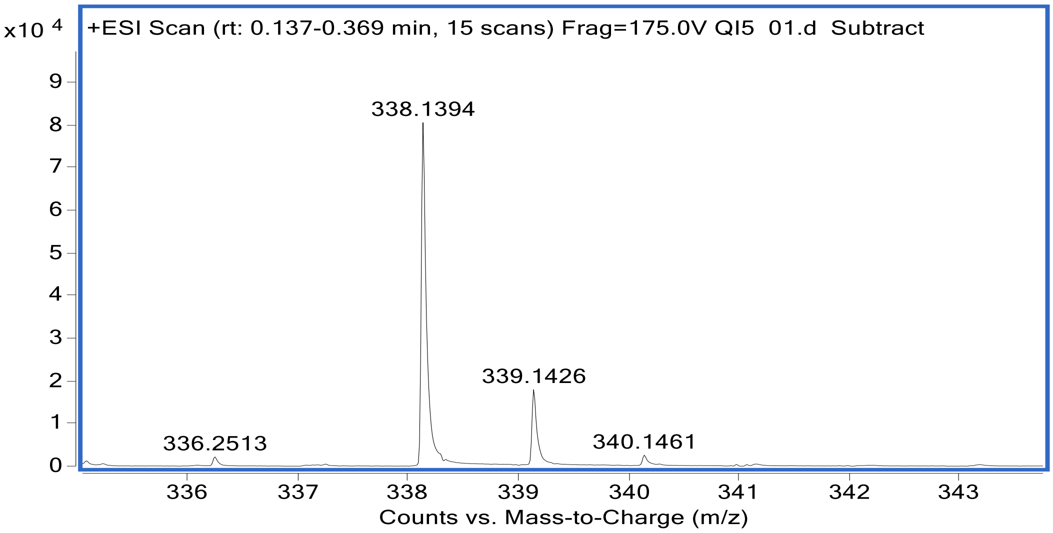

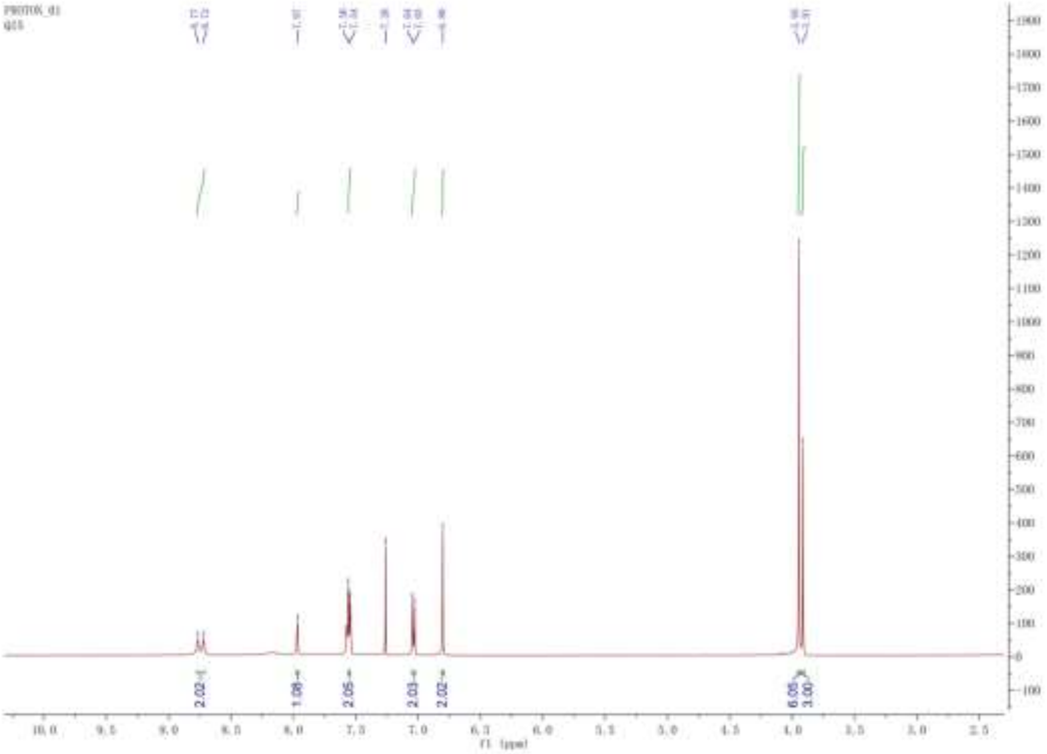

Information Classification: General

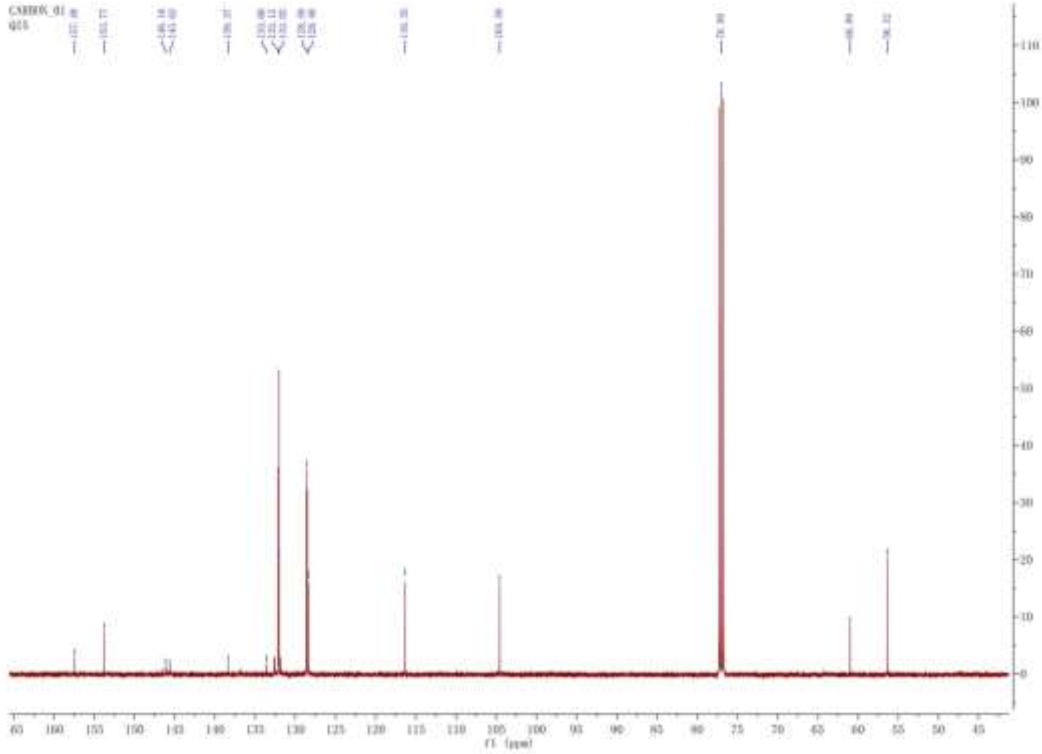

f1 (ppm)

*3-(4-fluorophenyl)-5-(3,4,5-trimethoxyphenyl)pyridine (**10m**)*

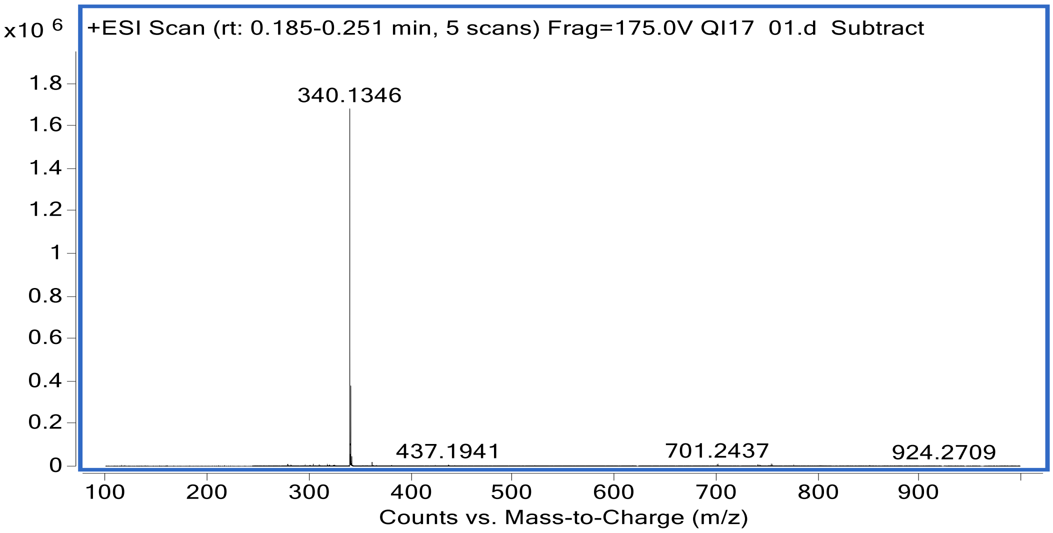

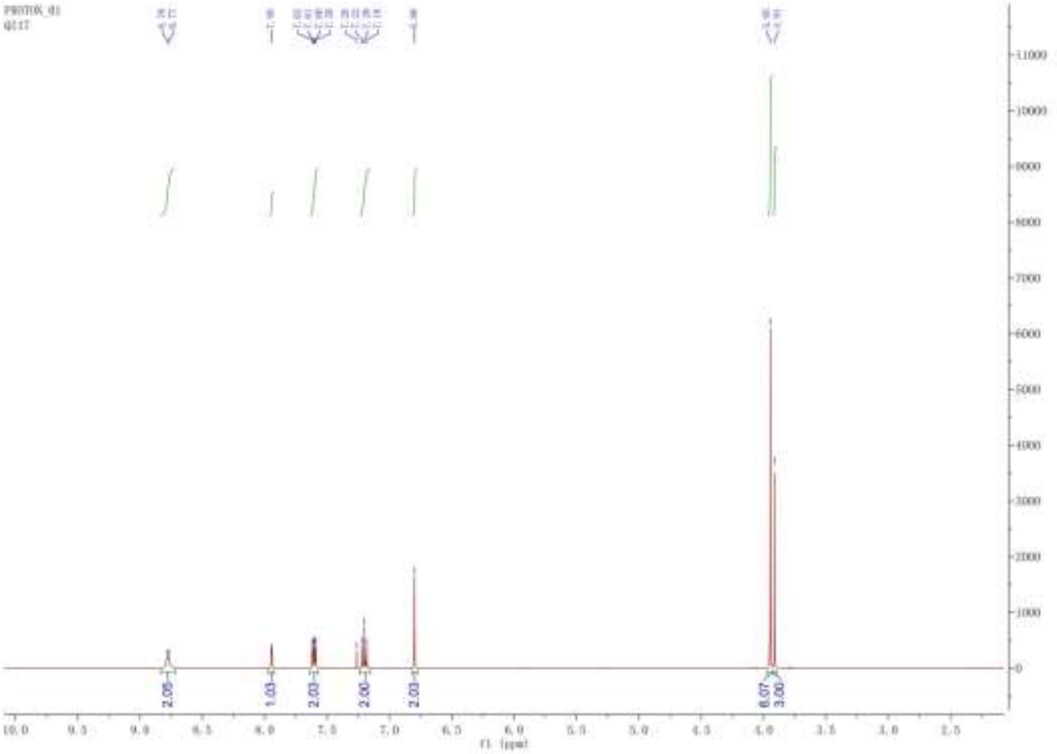

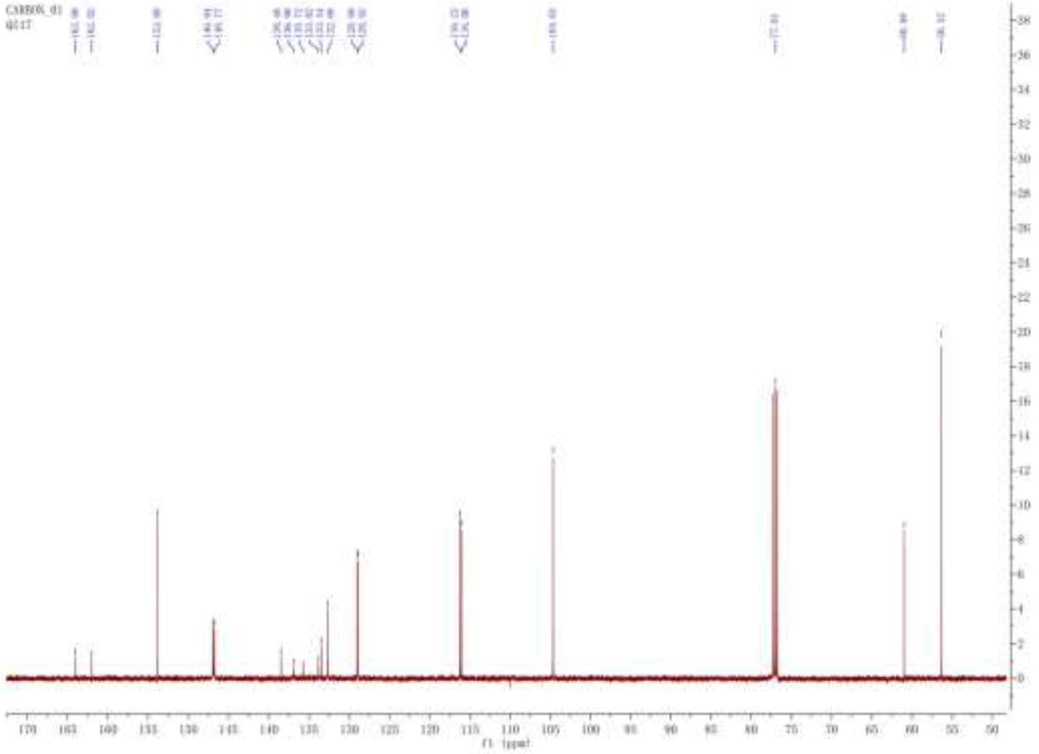

*3-(4-chlorophenyl)-5-(3,4,5-trimethoxyphenyl)pyridine (**10n**)*

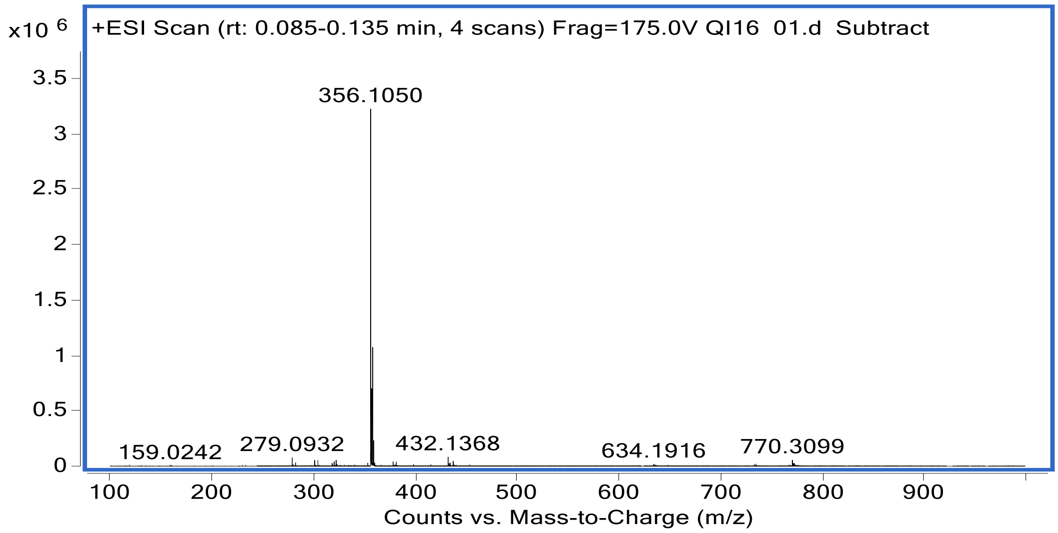

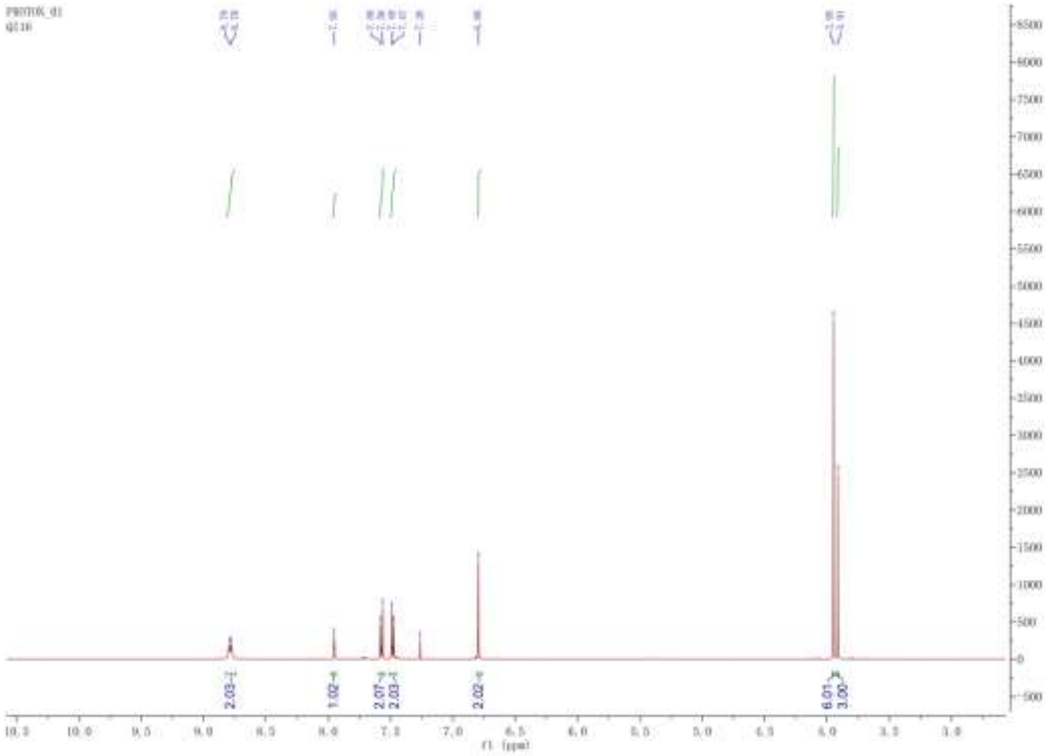

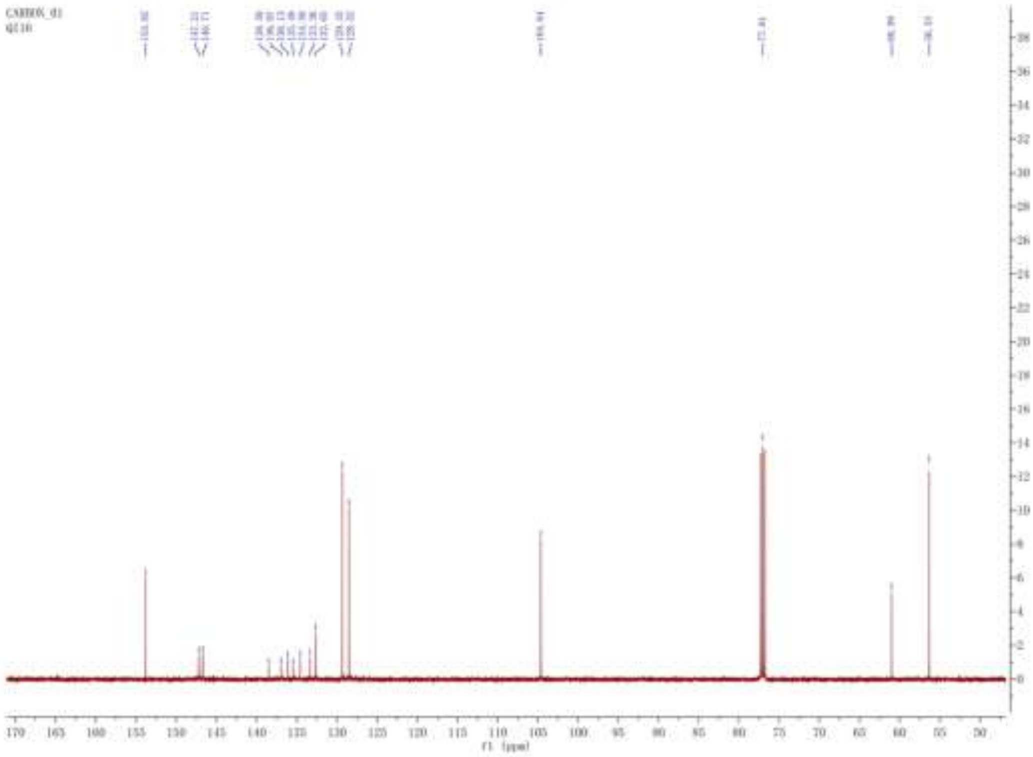

Information Classification: General

*3-(4-bromophenyl)-5-(3,4,5-trimethoxyphenyl)pyridine (**10o**)*

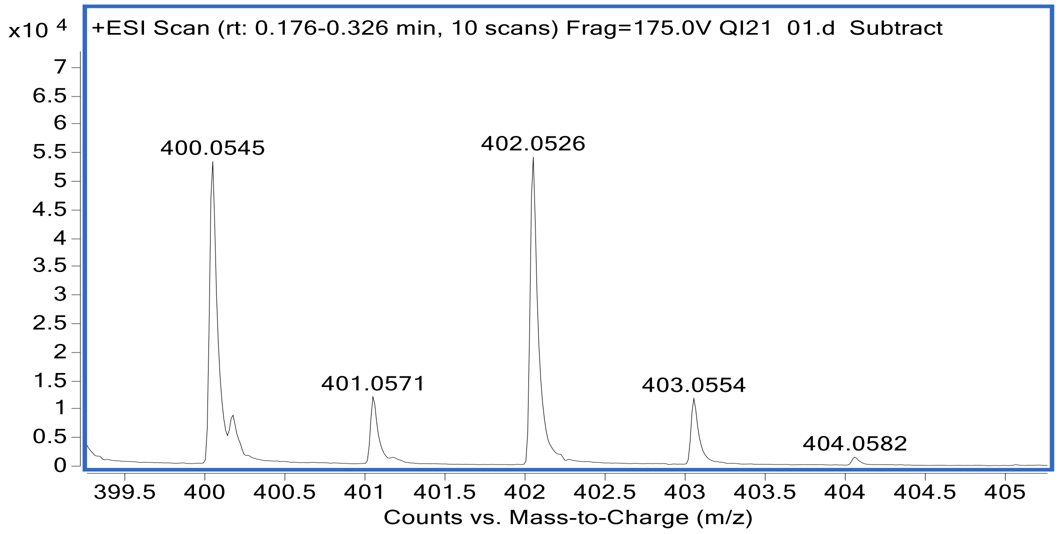

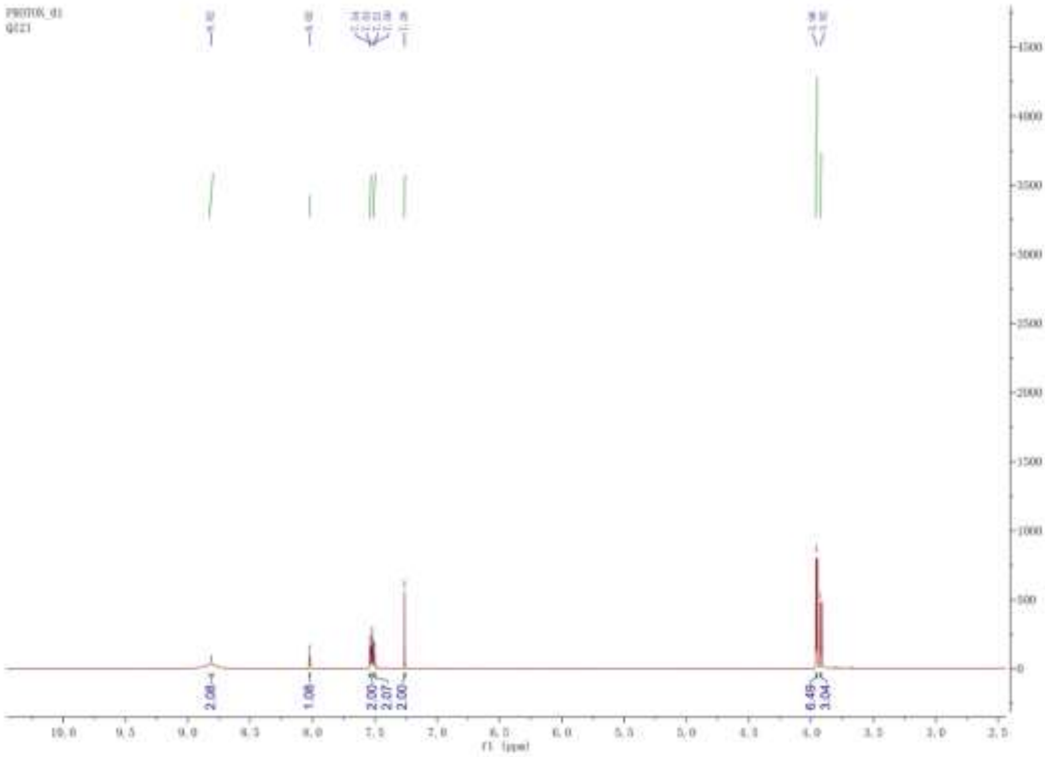

Information Classification: General

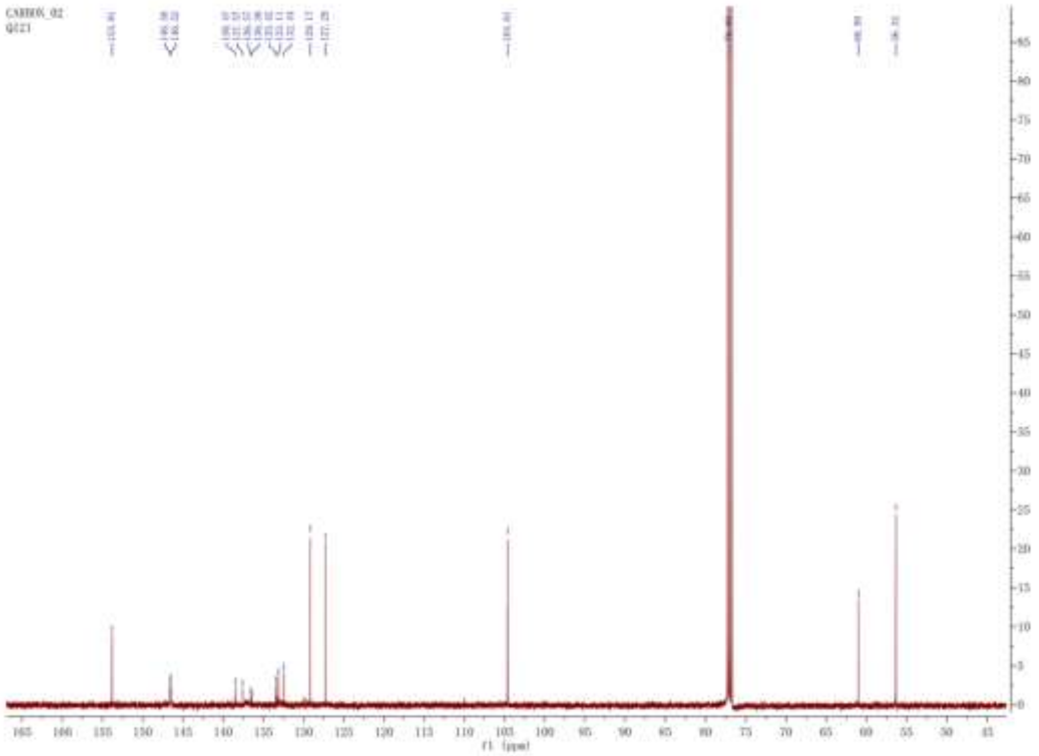

*3-(4-nitrophenyl)-5-(3,4,5-trimethoxyphenyl)pyridine (**10p**)*

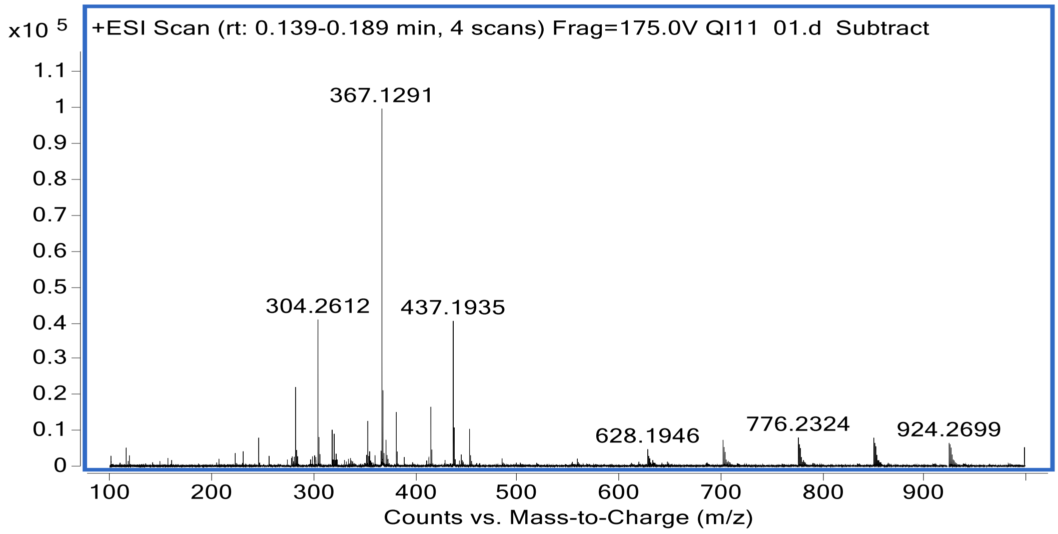

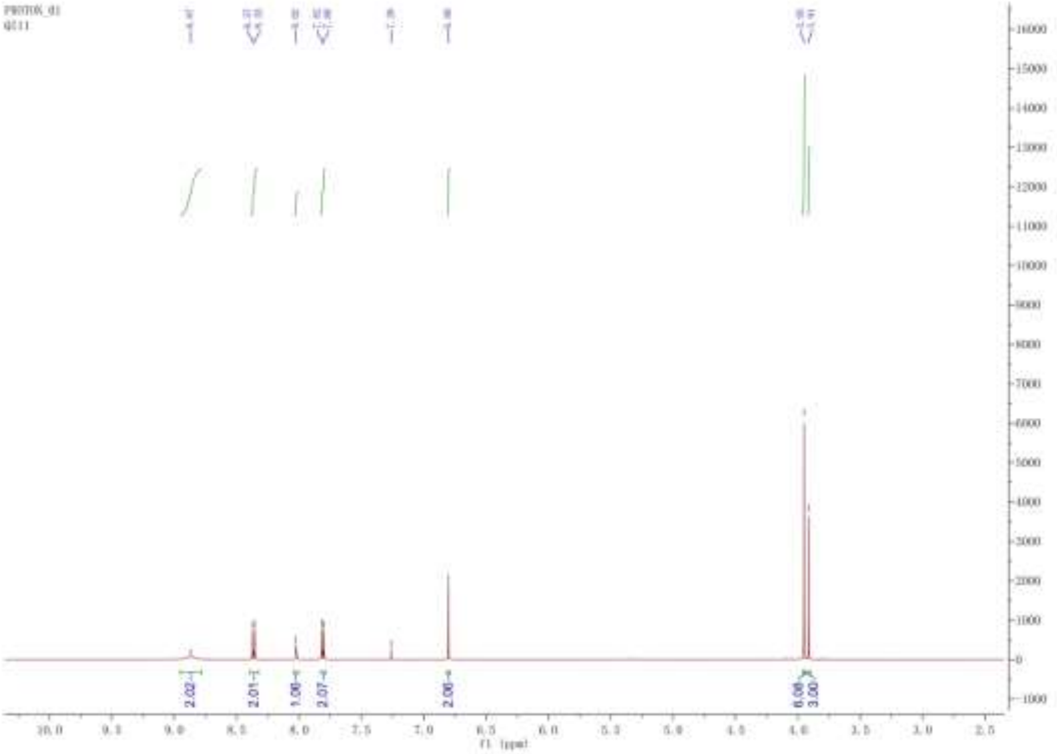

Information Classification: General

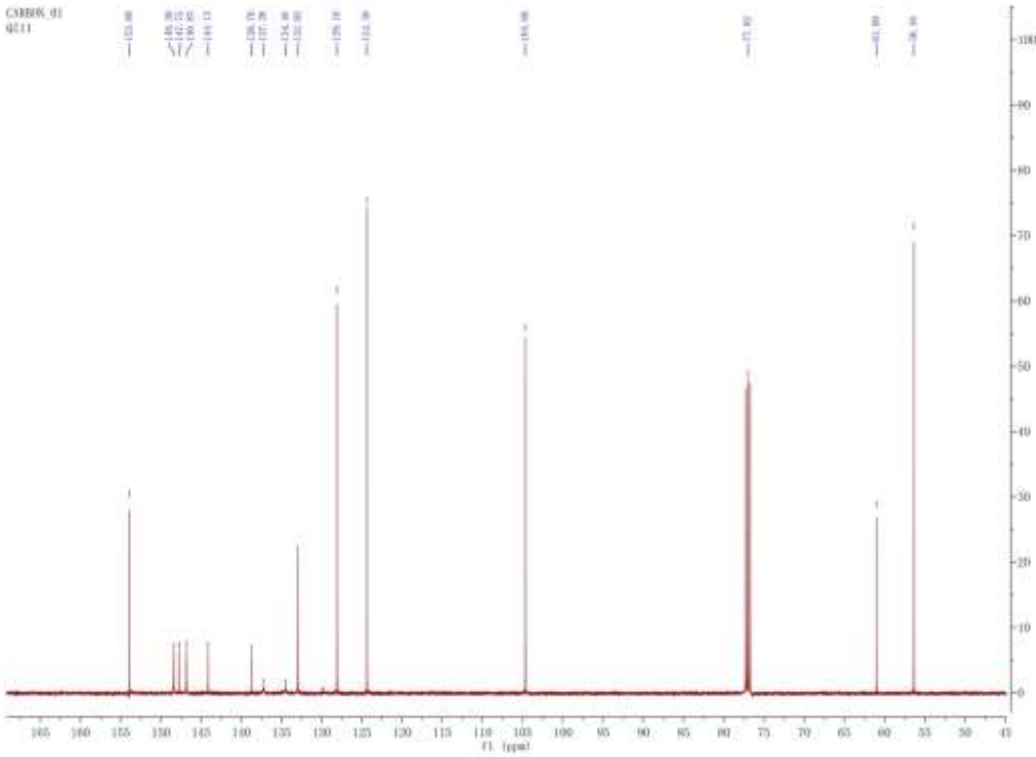
CARBON_01
f1 (ppm)

3-(thiophen-3-yl)-5-(3,4,5-trimethoxyphenyl)pyridine (***10q***)

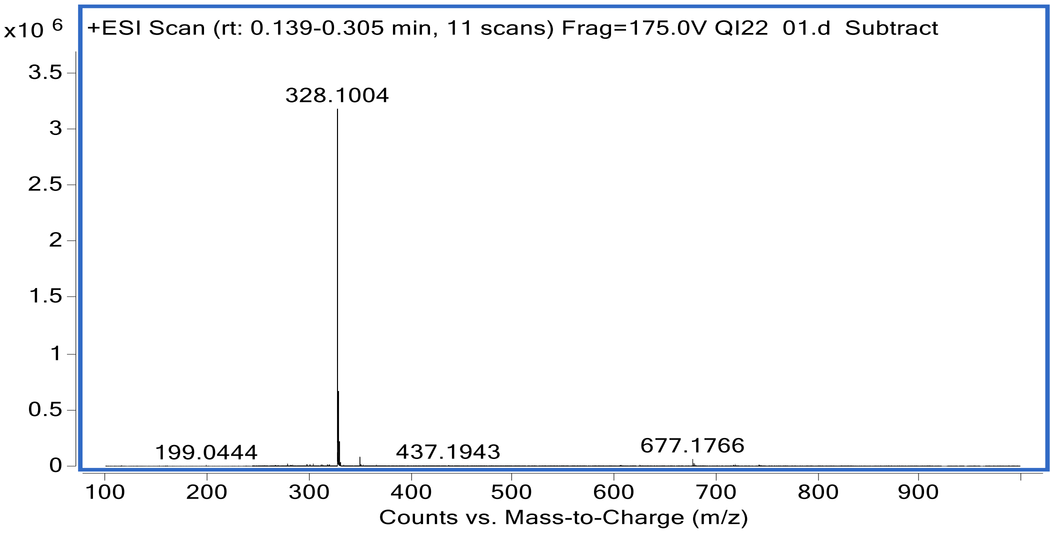

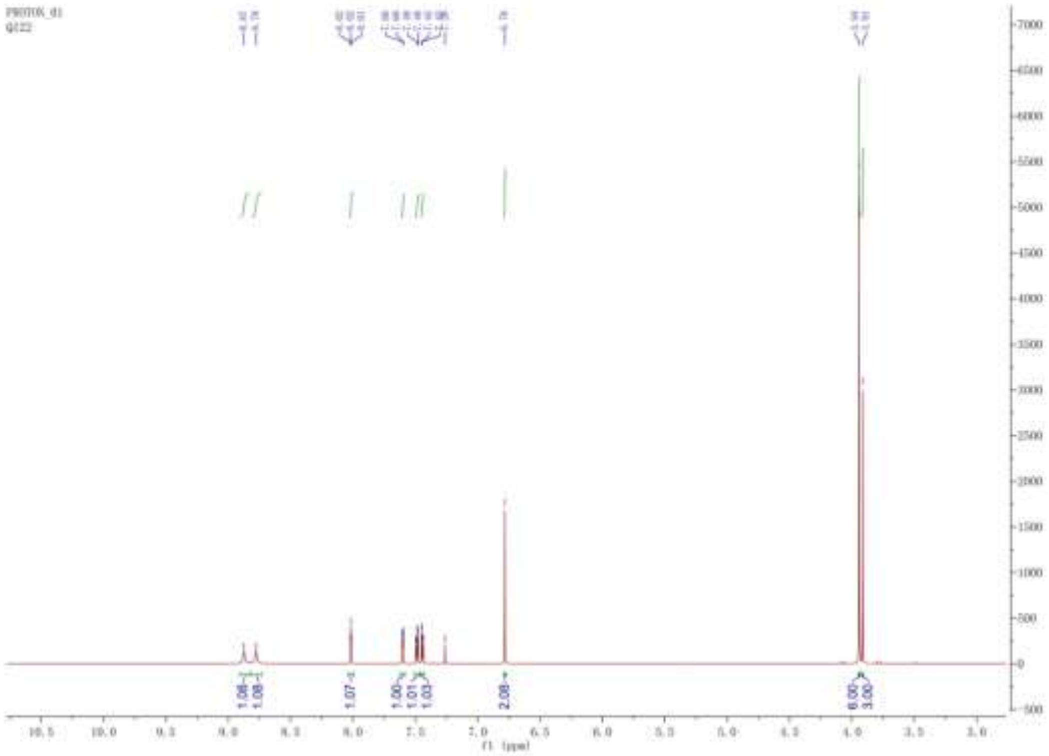

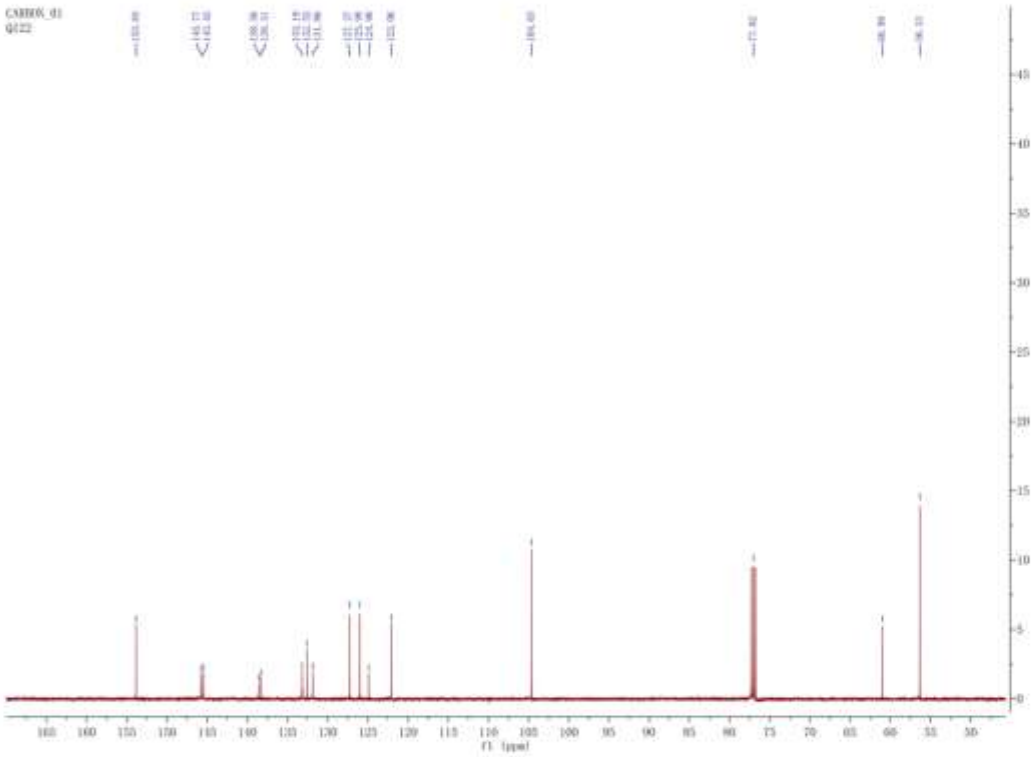

Information Classification: General

*5-(3,4,5-trimethoxyphenyl)-3,3'-bipyridine (**10r**)*

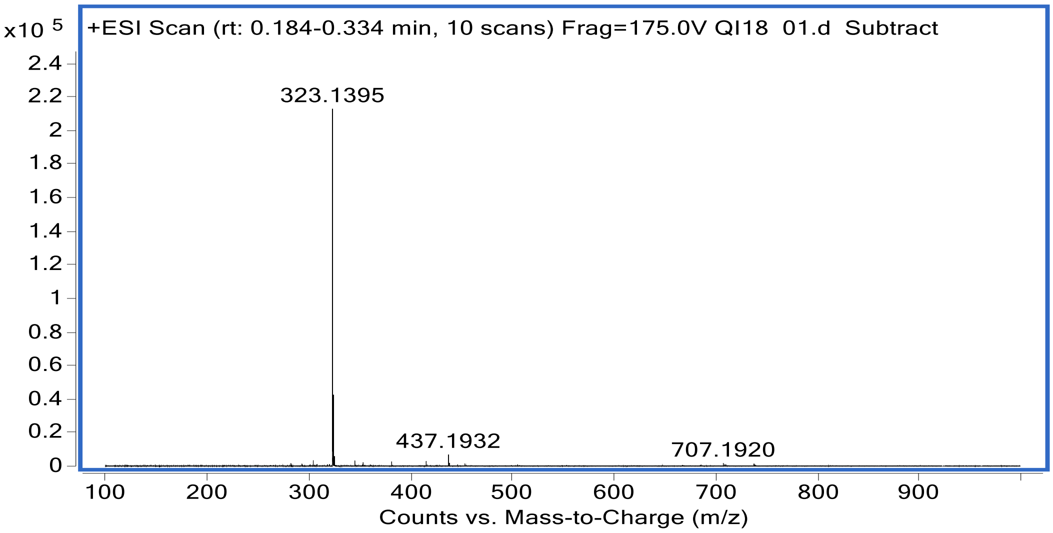

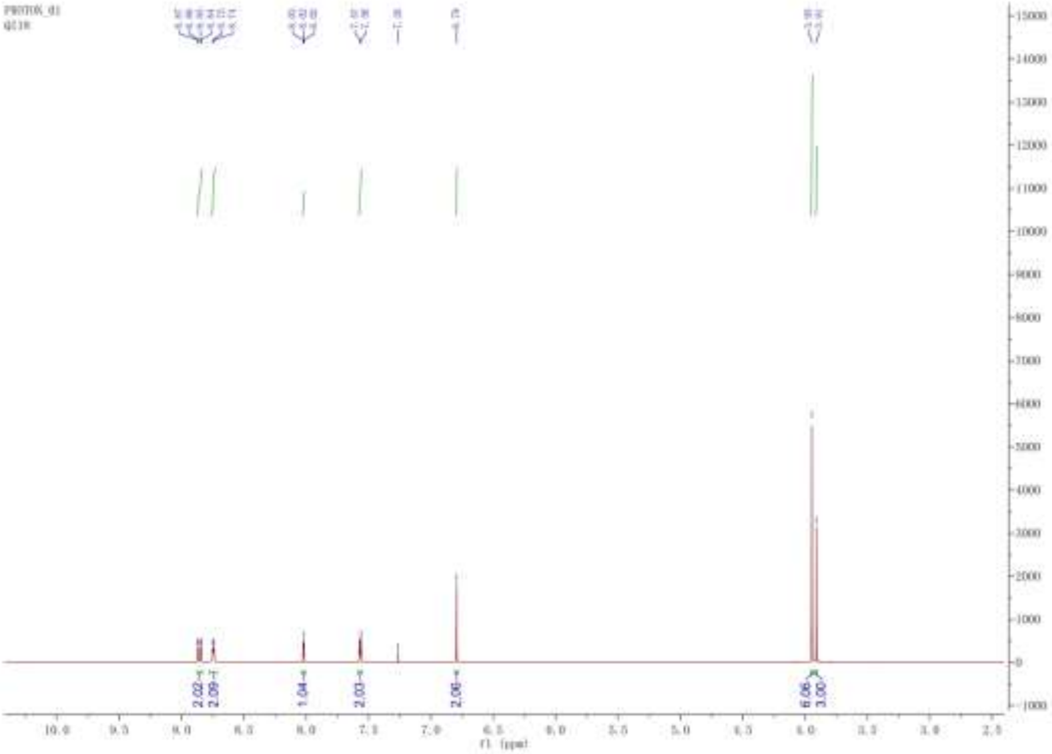

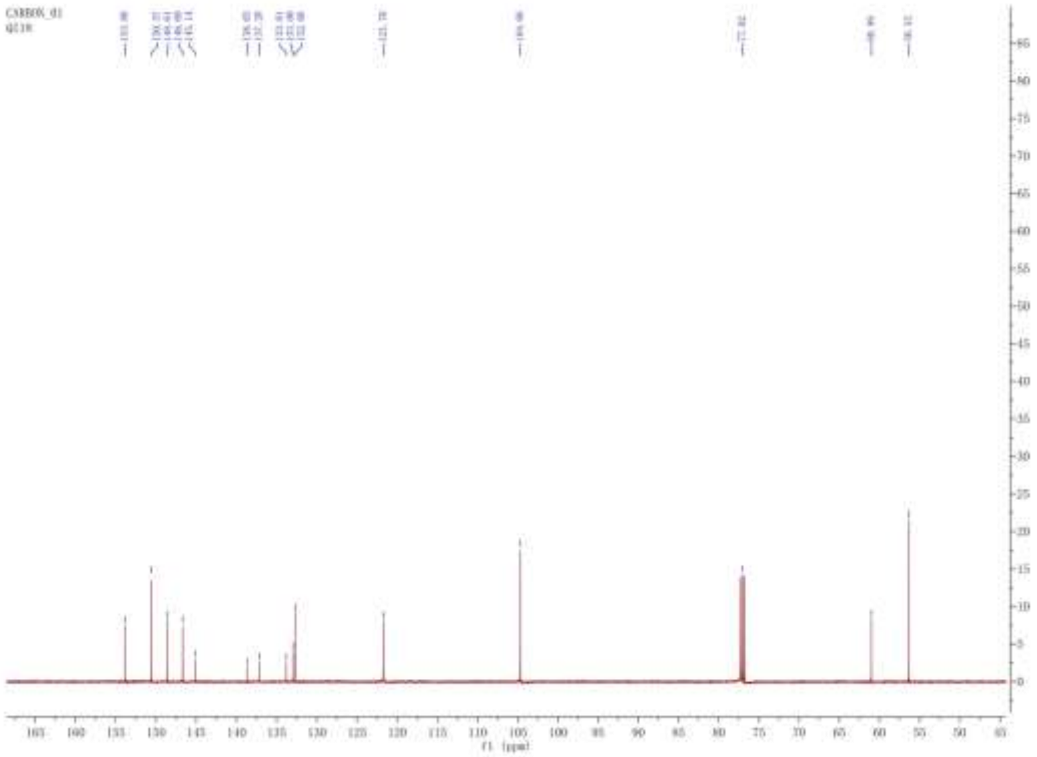
CARBON_01
f1 (ppm)

*5-(3,4,5-trimethoxyphenyl)-3,4'-bipyridine (**10s**)*

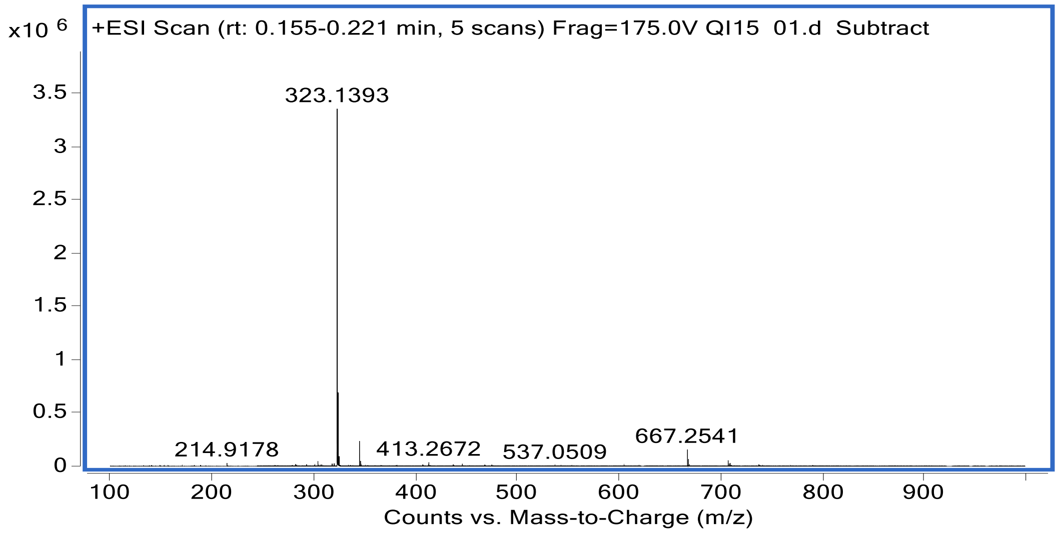

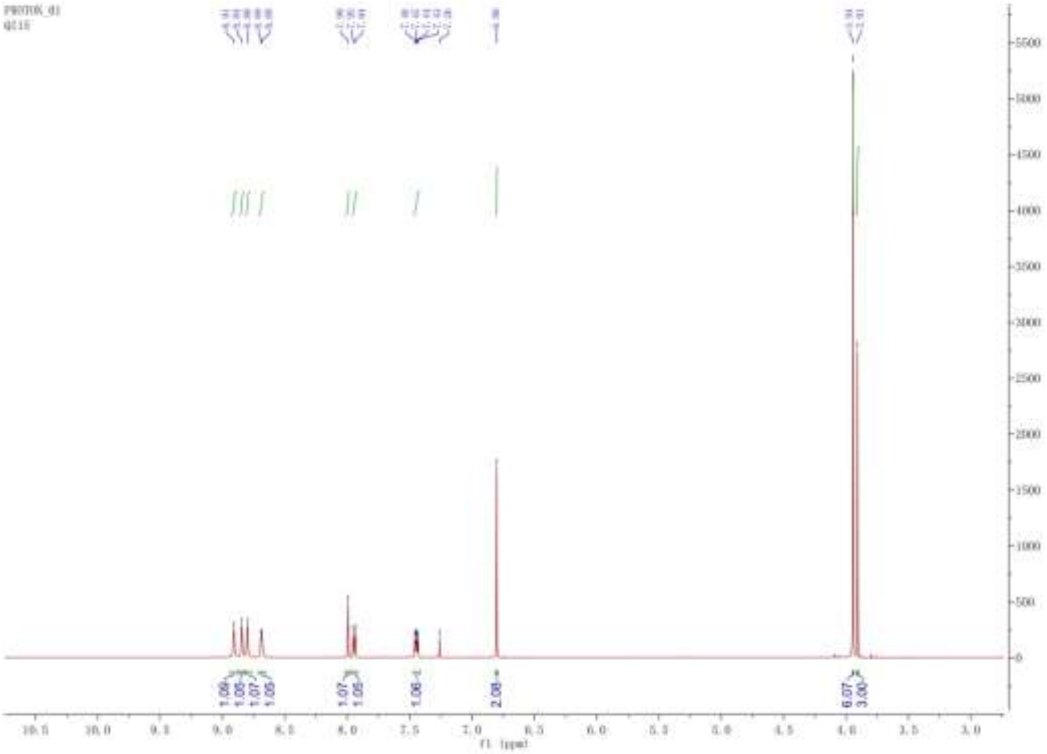

Information Classification: General

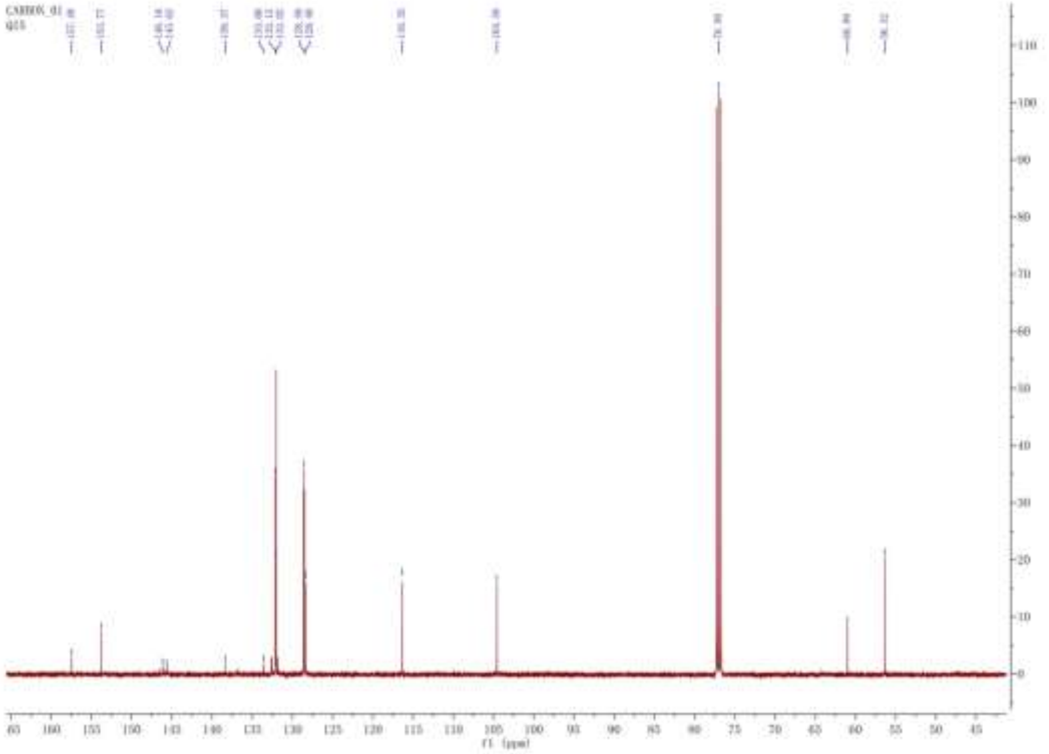
f1 (ppm)

*4-(5-(3,4,5-trimethoxyphenyl)pyridin-3-yl)-1H-indole (**10t**)*

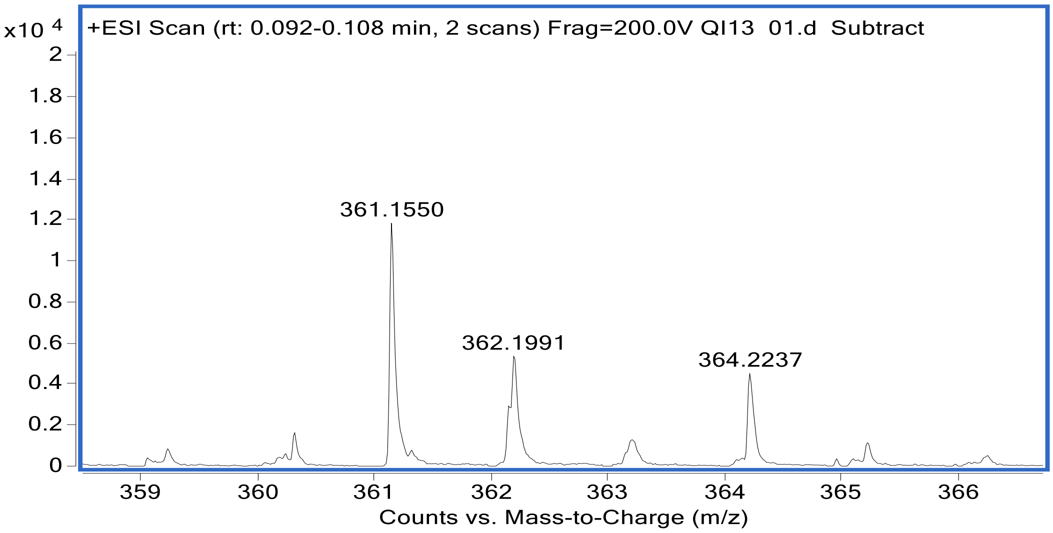

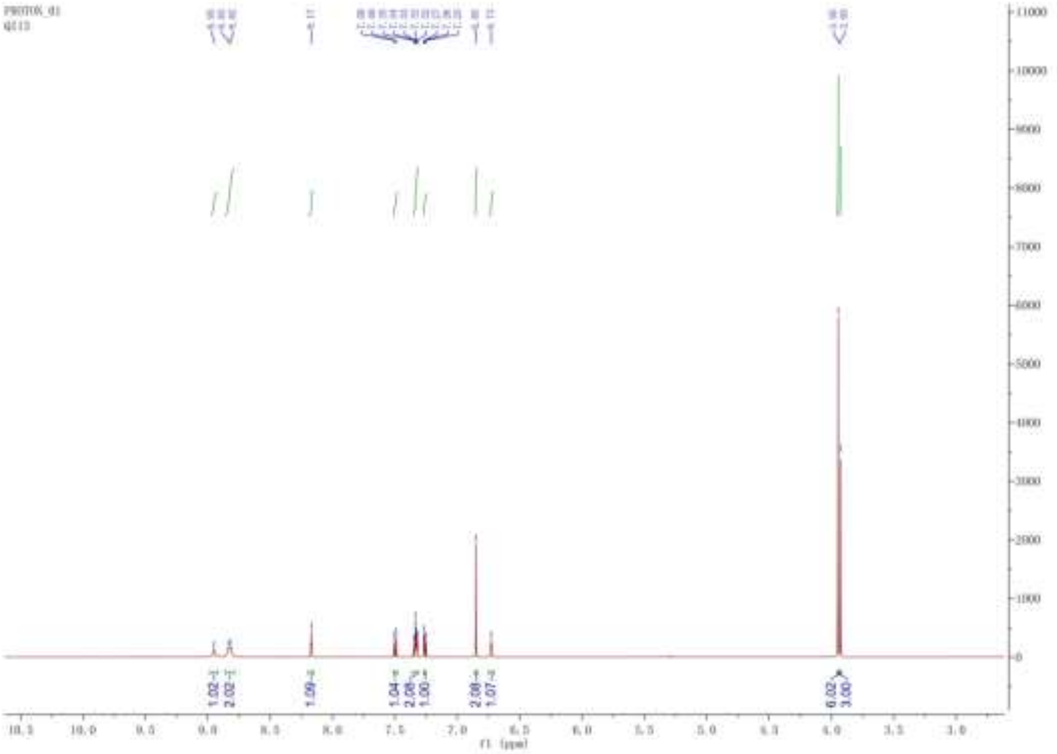

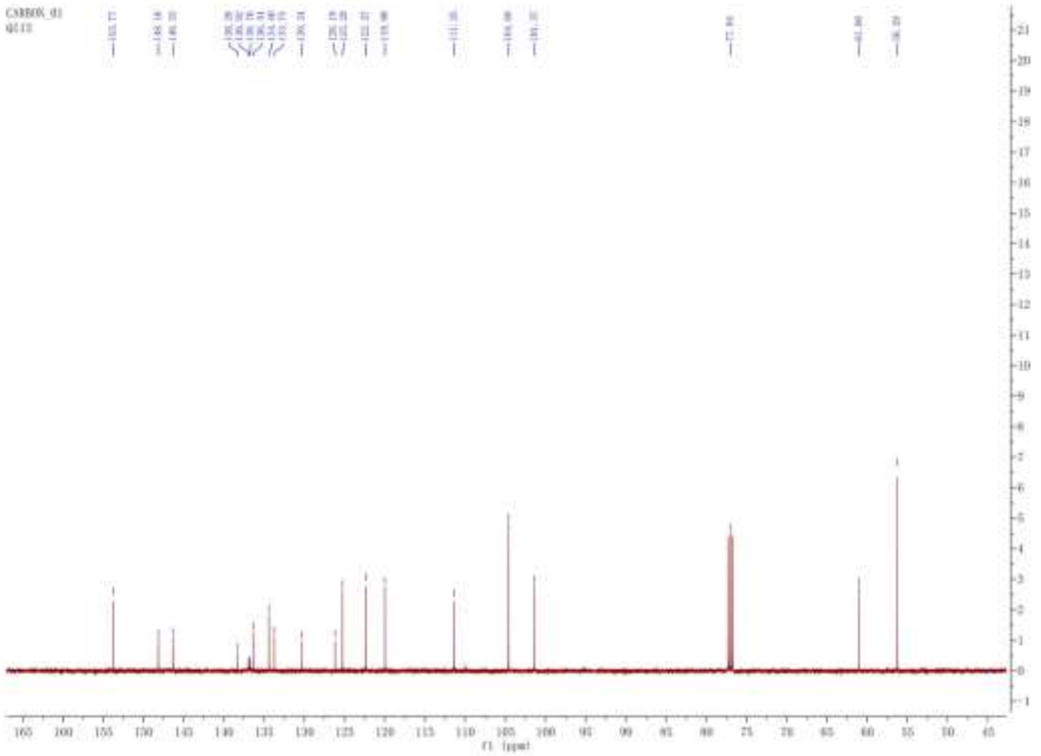

3-(naphthalen-2-yl)-5-(3,4,5-trimethoxyphenyl)pyridine (***10u***)

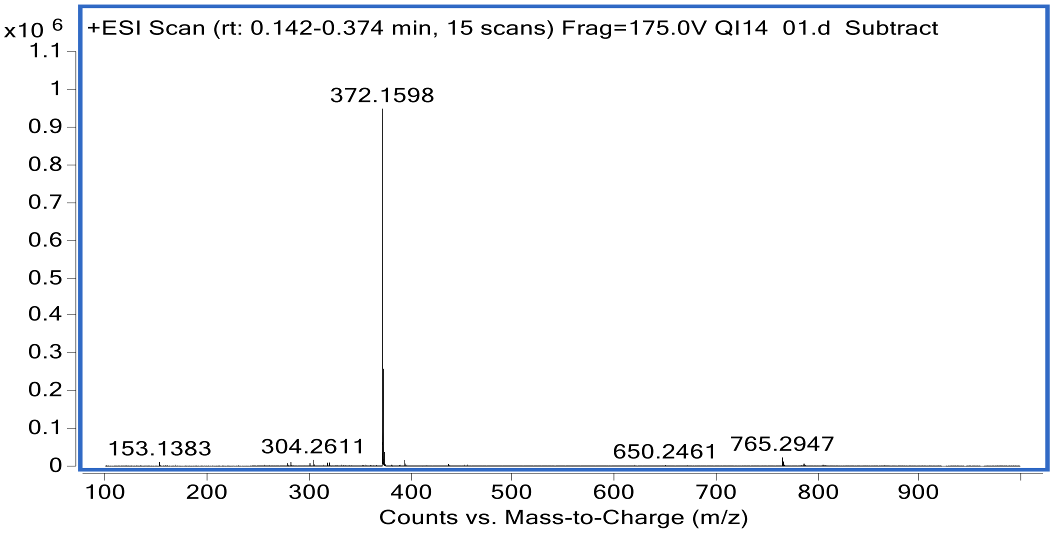

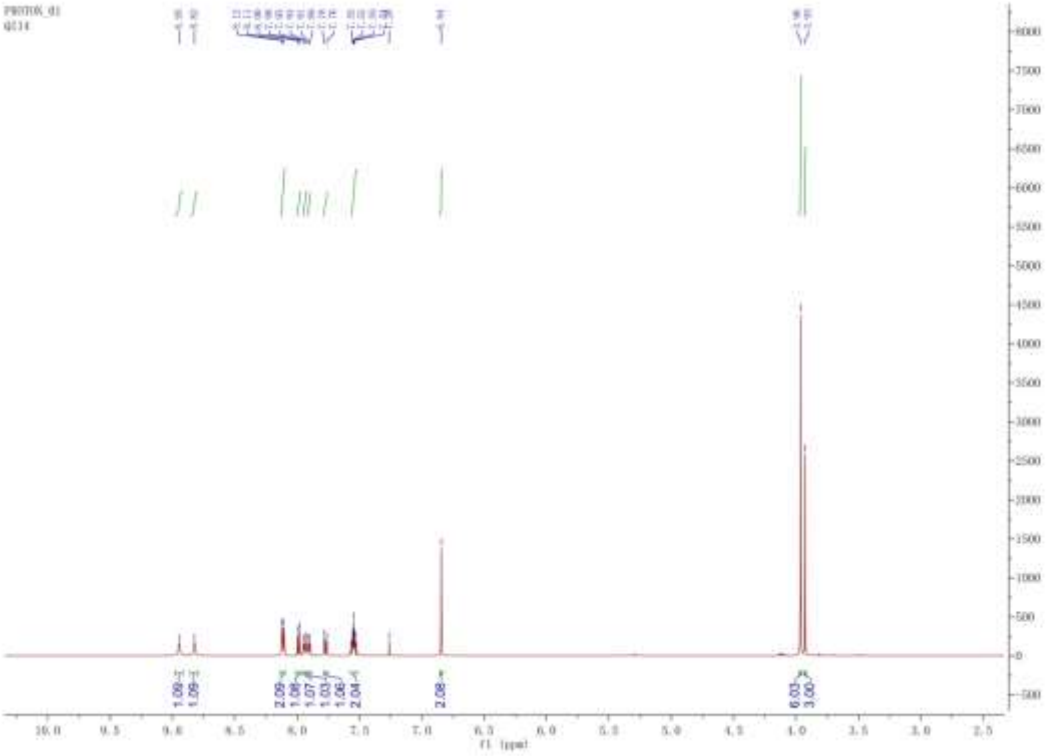

Information Classification: General

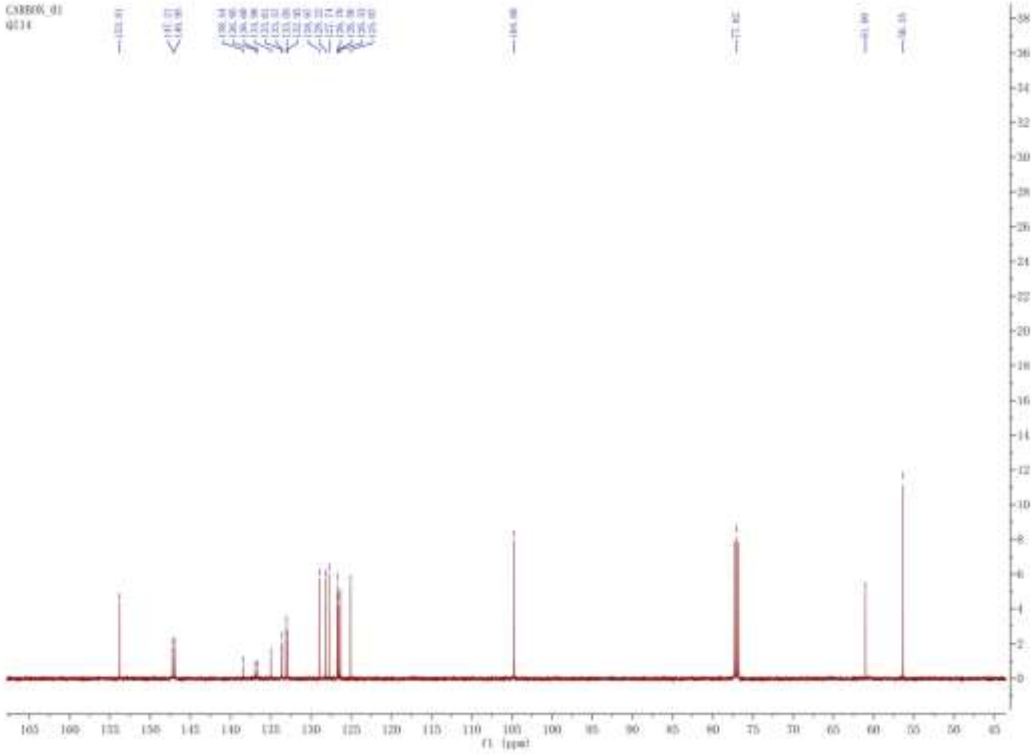
CARBON_01
f1 (ppm)
